# High-throughput capture of transcription factor-driven epigenome dynamics using PHILO ChIP-seq

**DOI:** 10.1093/nar/gkae1123

**Published:** 2024-11-26

**Authors:** Aanchal Choudhary, Moonia Ammari, Hyuk Sung Yoon, Mark Zander

**Affiliations:** Waksman Institute of Microbiology, Department of Plant Biology, Rutgers, The State University of New Jersey, Piscataway, NJ 08854, USA; Waksman Institute of Microbiology, Department of Plant Biology, Rutgers, The State University of New Jersey, Piscataway, NJ 08854, USA; Waksman Institute of Microbiology, Department of Plant Biology, Rutgers, The State University of New Jersey, Piscataway, NJ 08854, USA; Waksman Institute of Microbiology, Department of Plant Biology, Rutgers, The State University of New Jersey, Piscataway, NJ 08854, USA

## Abstract

Assessing the dynamics of chromatin features and transcription factor (TF) binding at scale remains a significant challenge in plants. Here, we present PHILO (Plant HIgh-throughput LOw input) ChIP-seq, a high-throughput ChIP-seq platform that enables the cost-effective and extensive capture of TF binding and genome-wide distributions of histone modifications. The PHILO ChIP-seq pipeline is adaptable to many plant species, requires very little starting material (1mg), and provides the option to use MNase (micrococcal nuclease) for chromatin fragmentation. By employing H3K9ac PHILO ChIP-seq on eight *Arabidopsis thaliana* jasmonic acid (JA) pathway mutants, with the simultaneous processing of over 100 samples, we not only recapitulated but also expanded the current understanding of the intricate interplay between the master TFs MYC2/3/4 and various chromatin regulators. Additionally, our analyses brought to light previously unknown histone acetylation patterns within the regulatory regions of MYC2 target genes in *Arabidopsis*, which is also conserved in tomato (*Solanum lycopersicum*). In summary, our PHILO ChIP-seq platform demonstrates its high effectiveness in investigating TF binding and chromatin dynamics on a large scale in plants, paving the way for the cost-efficient realization of complex experimental setups.

## Graphical Abstract

**Graphical Abstract:**
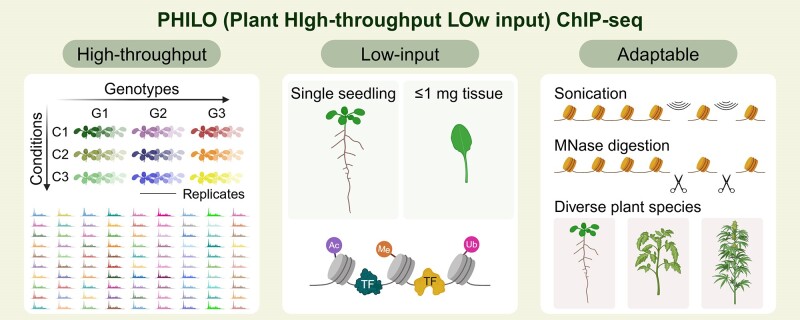

## Introduction

The plant epigenome plays a crucial role as a regulatory framework, integrating both developmental signals and environmental cues into spatiotemporal-specific gene regulatory networks (1). In a broad sense, the epigenome encompasses not only all chemical modifications of DNA and histone proteins but also other features that control gene expression, including DNA binding of transcription factors (TFs) and chromatin regulators, chromatin accessibility, 3D chromatin conformation, nucleosome positioning and long non-coding RNAs (2). Over the past two decades, numerous next-generation sequencing-based techniques were developed to investigate a wide range of epigenomic characteristics at the genomic level. Instances include MethylC-seq, which examines DNA methylation, ATAC-seq, which evaluates chromatin accessibility, and Hi-C, which delves into the three-dimensional structure of the genome (3–5). TF DNA binding as well as the distribution of histone modifications and variants, is typically obtained through Chromatin ImmunoPrecipitation (IP) analysis coupled with next-generation sequencing (ChIP-seq), CUT&RUN (Cleavage Under Targets and Release Using Nuclease) or CUT&Tag (Cleavage Under Targets and Tagmentation) (6–9).

According to ChIP-Hub, ChIP-seq is by far the most prominent platform in plant research with over 6000 reported experiments derived from more than 40 plant species since 2022 (10). However, only one-third of these have true biological replicates (10). The ENCODE and modENCODE consortia highly recommend at least two biological replicates for ChIP-seq since it can suffer from large experimental variation (11). Fragment extraction heterogeneities that arise from chromatin isolation, fragmentation through sonication, epitope-based IP and DNA precipitation are the main drivers of experimental variation (12). Another potential issue can be the modest dynamic range of histone modification occupancies and TF binding when compared to gene expression. In a diploid plant species, not considering endoreduplication events, the two histone H3 dimers of two + 1 nucleosomes at any given gene can only have post-translational modifications on maximal four histone tails. The principle of a modest dynamic range also applies to TF binding, even though predicting the actual number of detectable TF molecules bound to a regulatory region is considerably challenging due to several unknown factors, such as numbers of bound motifs, DNA residency times, TF stability and epitope accessibility. The low dynamic range, in combination with experimental variability among replicates, is less critical for qualitative ChIP-seq outputs, such as simply determining the presence or absence of a TF-binding peak or a specific histone domain. It can, however, pose significant challenges for assessing quantitative variation such as stimulus-induced changes in histone occupancies or TF binding.

A straightforward approach to mitigate experimental variability is to simultaneously analyze all ChIP-seq replicates. This can result in a significant rise in sample numbers, especially in complex experiments that involve multiple variables such as genotypes, time points, treatments and profiled features (such as histone modifications and/or TF binding). Thus, numerous high-throughput ChIP-seq platforms have been developed in various species ranging from HT-ChIP-seq (high-throughput Chromatin Immunoprecipitation), RELACS (restriction enzyme-based labeling of chromatin *in situ*) ChIP-seq and high-throughput ChIPmentation in mammals (13–15) to Bar-ChIP-seq (barcoded high-throughput ChIP-Seq) in yeast (16). There is currently no high-throughput platform available for plant tissues, primarily due to substantial sample volumes inherent in established plant ChIP-seq protocols (17,18).

**Figure 1.**
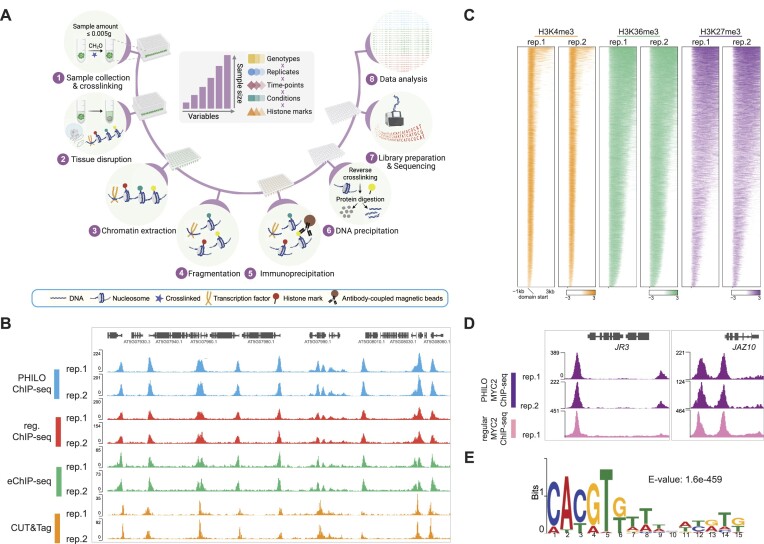
Overview of the PHILO ChIP-seq pipeline. (**A**) Schematic overview of the PHILO ChIP-seq pipeline with numbers indicating major steps of the protocol. The rapid increase in sample numbers with increasing numbers of experimental variables is also illustrated in the middle panel. Illustration was generated with BioRender. (**B**) Genome browser shows H3K9ac occupancy in two biological replicates of untreated 10-day-old *Arabidopsis* Col-0 seedlings determined by PHILO ChIP-seq, regular ChIP-seq, eChIP-seq, and CUT&Tag. (**C**) Heatmaps show H3K4me3-, H3K36me3- and H3K27me3-marked histones in two PHILO ChIP-seq replicates. (**D**) Genome browser shows DNA binding of MYC2 at two example genes (*JR3*, *JAZ10*) in two biological PHILO ChIP-seq replicates and one regular MYC2 ChIP-seq dataset that is derived from Zander et al. (54). (**E**) Enriched binding motif in the top500 MYC2 PHILO ChIP-seq derived MYC2 summits. Top 500 MYC2 summits results from a comparison of untreated and 2 h JA-treated *myc2 MYC2:MYC2-FLAG* seedlings.

Here, we introduce PHILO (Plant HIgh-throughput LOw input) ChIP-seq, a user-friendly and cost-effective high-throughput ChIP-seq platform designed to accommodate processing of over 100 samples in parallel, from tissue collection and disruption to IP and library preparation (Figure 1A). PHILO ChIP-seq represents a significant advancement in terms of scalability, simplicity, lower costs and reduced material requirements compared to existing methods. Not only did we employ PHILO ChIP-seq to evaluate the regulatory influence of individual components within an entire plant hormone pathway, but we also unveiled a novel stimulus-specific histone acetylation pattern surrounding TF-binding sites in *Arabidopsis* and tomato, termed Stimulus-Induced Enhancer Acetylation (SIENA) regions.

## Materials and methods

### Genetic material and growth conditions

All tested mutants and transgenic material used in this study are in the *Arabidopsis thaliana* Columbia-0 (Col-0) background. *myc2* (*jin1-8*) (19), *myc2 myc3 myc4* (20), *hac1-4* (21), *med25* (*pft1-2*) (22), *tpl tpr1 tpr4* (23), *hda6* (*axe1-5*) (24), *ninja-1* (25), *jazD (jaz decuple*) (26) and the *myc2 MYC2:MYC2-FLAG* line (27) were described previously. Seeds were surface sterilized with bleach, rinsed with water, and stratified in the dark for 2 days at 4°C. Seeds were planted on agar plates containing Linsmaier and Skoog (LS) medium (Phytotech Labs), supplemented with 1% sucrose (Sigma), and grown in a growth chamber (AR-41L3; Percival scientific) with a 16 h light/8 h dark cycle, at 19°C. Ten-day-old seedlings were treated with gaseous methyl jasmonate (MeJA; 95% purity; Sigma-Aldrich) in a closed transparent container for the respective times with MeJA applied to a Whatman paper (1 μl per 1 litre container volume). Lids of the agar plates were removed before the MeJA treatment. For the JA withdrawal (WD) (2 h JA + 2 h WD) time points, transparent containers were removed. After the respective treatments, tissue was rapidly collected, weighed (∼0.1 g), transferred into 1.1 ml tubes and subjected to crosslinking. Two-week-old Micro-Tom tomato (*Solanum lycopersicum*,*Sl*) and Otto II Boax hemp (*Cannabis sativa*) seedlings grown under a 16 h light/8 h dark cycle at 21°C were used for PHILO ChIP-seq analysis. Gaseous JA treatments of tomato seedlings were also conducted in a transparent container. Growth, handling and processing of hemp materials were conducted under the licenses for hemp growers (34_00011), hemp processors (34_00046) and hemp handlers (34_00047) issued to Rutgers University by the New Jersey Department of Agriculture.

### PHILO ChIP-Seq

#### Crosslinking

Unless otherwise indicated, all steps were performed with multichannel pipettes. We recommend preparing all buffers freshly. Samples were crosslinked in 500 μl 1% formaldehyde solution via vacuum infiltration at room temperature. Vacuum was applied for 10 min, released for 5 min and reapplied for an additional 10 min. Samples were centrifuged for 2 min at 2000 g. Supernatants were carefully discarded, and formaldehyde was quenched with 500 μl 0.25 M glycine solution under vacuum for 10 min. Crosslinked samples were rinsed with sterile water, thoroughly dried, frozen in liquid nitrogen and ground into a fine powder using Geno/Grinder tissue homogenizer.

#### Antibody-coupling

The following antibodies were used in this study: anti-H3K9ac (#39137, Active Motif), anti-H3K36me3 (#61021, Active Motif), anti-H3K27me3 (#39155, Active Motif), anti-H3K4me3 (#04–475, Millipore Sigma), FLAG (#F1804, Millipore Sigma) and anti-RNA polymerase II 8WG16 (#664906, Biolegend). IgG (#015–000-003, Jackson ImmunoResearch) served as a control. IPs were carried out in 96-well polymerase chain reaction (PCR) plates or PCR 8-tube strips with attached dome cap strips (USA Scientific). For each IP, 10 μl of Dynabeads Protein G (ThermoFisher Scientific) were washed thrice with 150 μl 1 × PBS buffer supplemented with 0.5% BSA. Each washing step consisted of 5 min rotating and subsequent capture of magnetic beads with a DynaMag-96 Side Magnet (ThermoFisher Scientific). After the last wash, beads were resuspended in 150 μl PBS-BSA buffer and the respective antibodies (0.5 μl of anti-H3K4me3, 1 μl of anti-H3K9ac, anti-H3K36me3 anti-H3K27me3 and IgG, 2 μl for anti-FLAG and anti-RNAPII) were added to the beads and incubated while rotating for at least 4 h at 4°C. Antibody-coupled beads were washed thrice with PBS-BSA buffer before sonicated chromatin was added (see below).

#### Chromatin extraction and sonication

For chromatin extraction, 250 μl of cold Buffer A (50 mM Tris-HCl pH7.5, 150 mM NaCl, 1 mM EDTA, 1% Triton X-100, 0.1% sodium deoxycholate, 1% SDS, 1x protease inhibitor cocktail (#04693132001, MilliporeSigma)) was added to each ground sample and samples were subsequently rotated for 15 min at 4°C. Homogenates were mixed with 750 μl of Buffer M (50 mM Tris-HCl pH7.5, 150 mM NaCl, 1 mM EDTA, 1% Triton X-100, 0.1% sodium deoxycholate, 1x protease inhibitor cocktail (#04693132001, MilliporeSigma)) and again rotated for 15 min at 4°C. Samples were then centrifuged at 2000 g for 10 min at 4°C, the supernatants were discarded and the pellets were carefully resuspended in 125 μl of Buffer A. For low input samples, exact weights of ground samples were rapidly measured on a fine scale before processing them using similar volumes of buffers. To conduct PHILO ChIP-seq with a specific number of *Arabidopsis* nuclei, purified nuclei were stained with 4,6-Diamidino-2-phenylindole (DAPI) and quantified with a hemocytometer. Nuclei were resuspended in 125 μl of Buffer A. Crude nuclei extracts were sonicated to 200–500 bp fragments in 0.5 ml tubes using a Bioruptor (Diagenode) with a 12 × 0.5 ml tube holder (24–36 cycles of 30 s ‘ON’ and 30 s ‘OFF’). We recommend determining the optimal cycle number in a pilot experiment. Most researchers have no access to a 96-well sonicator and depending on the experimental setup, sonication of samples must be carried out successively. To avoid time differences on ice between chromatin extraction and sonication among samples, we recommend splitting the experiment into sets of 12 samples and starting them successively delayed by 1 h per set. This 1 h delay among the sets is maintained until the de-crosslinking step which can be extended to 16 h or longer to merge all samples for parallel processing.

#### MNase fragmentation

As a liquid handling-only option, MNase (micrococcal nuclease) can be used for fragmentation instead of sonication. After incubating the homogenates with 750 μl of Buffer M and centrifugation at 2000 g for 10 min at 4°C, the pellets were carefully resuspended in 125 μl MNase buffer (50 mM sodium phosphate, 5 mm NaCl and 2.5 mm CaCl_2_). Around 4 U (units) of MNase (#2910A, Takara) was used per sample with starting amounts around 50–100 mg. For low amounts (5–25 mg) or single seedlings, 1 U MNase per sample is suitable, yielding 80–90% mono-nucleosomes. Samples were incubated for 15 min at 37°C and MNase was subsequently inactivated with 8 μl of 0.5 M EDTA.

#### Immunoprecipitation and DNA precipitation

Sonicated or MNase-digested lysates were centrifuged at full speed for 10 min at 4°C, and 70 μl of the supernatants that contain the sonicated chromatin were now added to the respective washed antibody-coupled magnetic beads. Around 210 μl of Buffer M was subsequently added and samples were incubated rotating overnight at 4°C. While the final 0.25% SDS concentration does not impact the IP with the antibodies employed in this study, it is advisable to assess the appropriate SDS concentration for each antibody separately. Beads were sequentially washed with low-salt buffer (50 mM Tris-HCl pH 7.5, 150 mM NaCl, 2 mM EDTA, 0.5% Triton X-100) (twice), high-salt buffer (50 mM Tris-HCl pH 7.5, 500 mM NaCl, 2 mM EDTA, 0.5% Triton X-100) (twice) and wash buffer (50 mM Tris-HCl pH 7.5, 50 mM NaCl, 2 mM EDTA) (twice). The immunoprecipitated protein-DNA complexes were eluted from beads by incubating them shaking at 65°C in 35 μl elution buffer (50 mM Tris-HCl pH 7.5, 10 mM EDTA, 1% SDS) for 15 min. After a quick spin, samples were placed on a magnetic rack for 1 min before supernatants were carefully transferred into new tubes. Remaining beads were resuspended again in 35 μl elution buffer and incubated shaking for 15 min at 65°C. Supernatants were then combined with the previous eluate and the resulting 70 μl eluates were now incubated for at least 6 h at 65°C followed by protein digestion with 1 μl proteinase K for 2 h at 55°C. For DNA precipitation, 70 μl of Phenol:Chloroform:Isoamyl Alcohol (25:24:1) was added to each sample and subsequently thoroughly vortexed for at least 20 s. After 15 min of centrifugation at 4000 g, approximately 70 μl of the upper aqueous phase was carefully transferred into new tubes. A stepwise transfer of smaller volumes is recommended. Around 2 μl of glycogen, 8 μl of 5 M NaCl, and 175 μl of absolute alcohol were then added to each sample before they were thoroughly mixed and stored for at least 3 h at −80°C. Samples were then centrifuged at 4000 g for 1 h at 4°C. Supernatants were carefully removed and 200 μl 70% ethanol was added to each sample. After centrifugation at 4000 g for 10 min at 4°C, the supernatant was carefully removed again. We recommend including a quick spin before removing the last microliters as the pellet is very small and can be easily lost. Switching to a 10 μl single-channel pipette is highly recommended. Precipitated DNA is finally resuspended in 50 μl sterile water.

#### Library preparation

Sera-Mag Solid Phase Reversible Immobilization beads were prepared as previously reported and used for library preparation (28). Depending on the experimenter's preference, library preparation can be carried out in 96-well PCR plates or PCR 8-tube strips with attached dome cap strips. The initial double-size selection of chipped DNA was carried out as previously reported with some modifications (17). Around 37.5 μl of Sera-Mag beads were added to each 50 μl chipped DNA sample. Samples were vigorously vortexed and incubated at room temperature for 10 min. Afterward, samples were placed on a magnetic rack for 5 min and supernatants were transferred to new PCR tubes. Around 62.5 μl of Sera-Mag beads were added and samples were vigorously vortexed and incubated at room temperature for 10 min again. Samples were placed then on a magnetic rack for 5 min and supernatants were discarded. Beads were washed twice with 200 μl freshly prepared 75% ethanol. Every washing step involves a swift rinse of the beads while they are still on the magnetic rack. The supernatant is then removed following each rinse. After the second wash, it is advisable to briefly spin the samples and return them to the magnetic rack to remove any remaining ethanol. Beads were dried on a ThermoMixer C with a SmartBlock PCR 96 (Eppendorf) for 3–5 min at 37°C and then resuspended in 19 μl resuspension buffer (10 mM Tris-Cl, pH 8.5). After vigorous vortexing, samples were incubated at room temperature for 10 min and beads were collected afterward on a magnetic rack for 2 min. Around 17.5 μl of supernatant was transferred to a new tube and 7.5 μl end repair master mix (5 μl of End Repair Reaction Buffer + 2.5 μl of End Repair Enzyme Mix from the NEBNext End Repair Module (New England Biolabs)) was added to each sample. Samples were incubated for 45 min at room temperature followed by a bead cleanup using 45 μl Sera-Mag beads and 22 μl resuspension buffer for elution.

Around 21 μl of supernatant was transferred to a new tube and 4 μl A-tailing master mix (2.5 μl NEBnext dA-Tailing Reaction Buffer + 1.5 μl Klenow Fragment (3′→5′ exo-) (New England Biolabs)) was added to each sample. Samples were incubated for 30 min at 37°C followed by a bead cleanup using 45 μl Sera-Mag beads and 21 μl resuspension buffer for elution. TruSeq DNA Unique Dual Indexes (UDIs) v2 (Illumina) were used for adapter ligation. Around 20.25 μl of supernatant was transferred to a new tube and 1 μl of a different Illumina UDI was added to each sample. Around 3.75 μl of ligation master mix (2.5 μl T4 DNA Ligase Reaction Buffer + 1.25 μl T4 DNA Ligase (New England Biolabs)) was added subsequently to each sample and samples were thoroughly mixed by pipetting up and down. After incubation overnight at 16°C, 25 μl ultrapure H2O was added to each sample followed by a double bead cleanup (first cleanup: 55 μl Sera-Mag beads and 50 μl resuspension buffer, second cleanup: 55 μl Sera-Mag beads and 11 μl resuspension buffer). Around 10 μl of supernatant was transferred to a new tube and 15 μl of enrichment PCR master mix (12.5 μl NEBNext High-Fidelity 2X PCR Master Mix (New England Biolabs) + 2.5 μl Primer cocktail (1.25 μl of 100mM primer P5 5′AATGATACGGCGACCACCGAGATCTAC and 25 μl of 100mM primer P7 5′CAAGCAGAAGACGGCATACGAGAT) was added. We also assessed three different PCR cycle numbers (10,12,14) and did not observe any major differences regarding uniquely aligned reads, FRiP scores, and the number of identified H3K9ac domains (Supplementary Figure S1A–C). Although 13–15 were used for most datasets in this study, 10 PCR cycles are sufficient. For the final bead cleanup, 28 μl Sera-Mag beads and 10 μl resuspension buffer for elution were used. Libraries were quantified with a Qubit 4 Fluorometer (ThermoFisher Scientific). Quality of the final pool was checked with a 5300 Fragment Analyzer System (Agilent) before sequencing on an Illumina NextSeq 500 sequencing system (Illumina) according to the manufacturer's guidelines. The sequencing depth that is required to capture most histone domains (Supplementary Figure S1D–F) was also tested. We found that 5 million reads per H3K9ac PHILO ChIP-seq sample are sufficient and that even 1M reads are enough to detect the majority of H3K9ac domains (Supplementary Figure S1D–F).

#### ChIP-seq

Regular ChIP-seq experiments were performed as previously described with minor modifications (17). ChIP-seq assays were conducted with 6 μl of anti-H3K9ac antibody (39137, Active Motif) for each sample. IgG (015–000-003, Jackson ImmunoResearch) served as the negative control. Dynabeads Protein G (ThermoFisher Scientific) were coupled for 4–6 h with respective antibodies and incubated overnight with equal amounts of sonicated chromatin. After washing the beads, samples were eluted, de-crosslinked, Proteinase K digested and DNA was subsequently precipitated. Libraries were prepared as described for PHILO ChIP-seq with some modifications. The double amount of master mixes for end repair, A-tailing, and enrichment PCR per sample was used. Sera-Mag bead and resuspension volumes increased accordingly. Libraries were single-end sequenced on an Illumina NextSeq 500 Sequencing system (Illumina).

#### RNA-seq

Total RNA of Col-0 seedlings was extracted using the RNeasy Plant Mini Kit (Qiagen). cDNA library preparation was carried out with a TruSeq Stranded mRNA Library Prep kit (Illumina) following the manufacturer's protocol.

#### enhanced ChIP-seq (eChIP-seq)

eChIP-seq experiments were performed in 1.5 ml microcentrifuge tubes as previously described using 0.1 g of Col-0 seedlings (29). Around 3 μl of anti-H3K9ac antibody (#39137, Active Motif) was used for each sample.

#### CUT&Tag

CUT&Tag assays were performed as previously described with minor modifications (8,30). Nuclei were extracted from *Arabidopsis* Col-0 seedlings (200 mg per sample) and resuspended in 450 μl Wash buffer (20 mM HEPES pH 7.5, 150 mM NaCl, 0.5 mM Spermidine, 1 × Protease inhibitor cocktail (#04693132001, MilliporeSigma)). For nuclei immobilization, 20 μl Concanavalin A-Coated Magnetic Beads (ConA) (#93569, Cell Signaling Technology) were washed with 200 μl Activation buffer (#93569, Cell Signaling Technology) thrice and resuspended in with 50 μl Activation buffer. Around 450 μl of nuclei were then incubated with 50 μl activated ConA beads for 30 min at 4°C under slow rotation. For antibody binding, bead-coupled nuclei were resuspended in 200 μl Dig-wash Buffer (20 mM HEPES pH 7.5; 150 mM NaCl, 0.5 mM Spermidine, 1 × Protease inhibitor cocktail (#04693132001, MilliporeSigma), 0.05% Digitonin, 2mM EDTA) and 1 μl of anti-H3K9ac antibody (#39137, Active Motif) overnight at 4°C. Primary antibodies were removed and nuclei were incubated with 2 μl of guinea pig anti-rabbit (#ABIN101961, Antibodies-Online) for 1 h at room temperature to increase IgG molecules at bound epitopes. Bead-coupled nuclei were then washed thrice with Wash buffer and resuspended in 100 μl Wash300 buffer (20 mM HEPES pH 7.5, 300 mM NaCl, 0.5 mM Spermidine, 1 × Protease inhibitor cocktail). CUTANA pAG-Tn5 (#15–1017, EpiCypher) was used for tagmentation. PCR enrichment and library DNA purification were carried out according to the manufacturer's protocol. Libraries were paired-end sequenced on an Illumina NextSeq 500 Sequencing system (Illumina).

### Sequencing data analysis

#### Quality control

To generate high-quality PHILO ChIP-seq sequencing data, a sequencing depth of 5 million reads per sample is typically sufficient. Increasing the depth beyond this does not significantly impact the signal-to-noise ratio, as measured by the FRiP (Fraction of fragments in peaks) score and has only a minor effect on the number of detectable histone domains (Supplementary Figure S1D–F). It is highly recommended to analyze at least two replicates to improve quality assessment. We evaluated PHILO ChIP-seq data quality using several metrics, including the mapping rate of uniquely aligned reads, FRiP scores, the number of detected peaks or domains and consistency across replicates. The ratio of uniquely mappable reads serves as an important indicator; we found that histone PHILO ChIP-seq using *Arabidopsis* tissues typically yields high-quality data when this ratio falls between 70 and 90%. FRiP scores, which quantify the signal-to-noise ratio by counting fragments within ChIP-seq peak or domain regions (11), were calculated using SAMtools (version 1.3.1) and BEDTools (version 2.25.0). High-quality PHILO ChIP-seq assays, usually have FRiP scores above 0.5 (Supplementary Figure S1E). Another important measure is the consistency in the number of detected histone domains or TF DNA binding peaks across replicates. A summary of all sequenced ChIP-seq, eChIP-seq, CUT&Tag and PHILO ChIP-seq samples, along with quality metrics, is provided in Supplementary Table S1.

#### PHILO ChIP-seq analysis

ChIP-seq, eChIP-seq, PHILO ChIP-seq and CUT&Tag sequencing reads were aligned to TAIR10 (*Arabidopsis thaliana*), SLM_r2.0.pmol (*Solanum lycopersicum* Micro-Tom (31)) and cs10 (*Cannabis sativa*) using Bowtie2 (version 2.4.1) (32,33). Genome-wide occupancies of histone modifications were determined with epic2 (version 0.0.52) using IgG samples as a control (34). For the identification of H3K9ac, H3K4me3, H3K36me3, and H3K27me3 domains, all three replicates were first merged and then subjected to epic2 analysis using IgG samples as a control. Domains that show a log2 fold change ≥1 when compared to an IgG control were used (*Arabidopsis*: 10569 H3K9ac domains, 15355 H3K4me3 domains, 11108 H3K36me3 domains, 5278 H3K27me3 domains; tomato 18163 H3K4me3 domains; hemp 14960 H3K4me3 domains) for visualization with heatmaps. For the comparison of H3K9ac PHILO ChIP-seq with publicly available H3K9ac ChIP-seq and CUT&Tag experiments, the following datasets were downloaded from GEO: (H3K9ac ChIP-seq: Zhou et al., 2024, GSE252996 (SRR27496336, SRR27496339) (35), Huang et al., 2021, GSE155502 (SRR13746647, SRR13746649) (36), Wang et al., 2017, GSE77394 (SRR3134765, SRR3134766) (37), Zhao et al., 2022, GSE183957 (SRR15853529, SRR15853529)) (38), (H3K9ac CUT&Tag: Liu et al., 2024, GSE243403 (SRR26087729)) (39). To identify MYC2 binding peaks and MYC2 peak summit regions, MACS2 (version 2.2.7.1) was used (40). Tomato MYC2 ChIP-seq data was previously published (41). Both tomato MYC2 ChIP-seq replicates (CRD029134, CRD029135) were used to identify MYC2 binding peaks with MACS2 (version 2.2.7.1) (40). Replicate CRD029134 is shown in IGV genome browsers in Figure 4E, F and Supplementary Figure S7B, C. To identify preferred binding motifs within the top 500 summit regions of two merged MYC2 ChIP-seq experiments, the MEME-ChIP analysis tool was employed (42). For peak annotation, ChIPseeker (Version 1.28.3) was used (43). The Database for Annotation, Visualization and Integrated Discovery was used to identify gene ontology enrichment in the MYC2 core gene set of 179 genes (44,45). ChIP and PHILO ChIP-seq replicates were merged, sorted and indexed with SAMtools (version 1.3.1) and BEDTools (version 2.25.0) (46,47). Heatmaps, aggregated profiles and correlation analyses of ChIP-seq, eChIP-seq, PHILO ChIP-seq and CUT&Tag data were carried out with deepTools (version 3.5.2) (48). To generate heatmaps visualizing PHILO ChIP-seq data, regions with a significant enrichment of a histone mark were identified with epic2 using merged replicates and merged IgG replicates as a control. For the quantification of H3K9ac levels in gene bodies and SIENA region of MYC2 core genes, the bigWigAverageOverBed tool executable from the UCSC genome browser was used for the indicated regions (49). The H3K9ac levels were determined by calculating the ratio of H3K9ac to IgG control. Negative values were set to 0.001. To facilitate visualization, the average value of all three 2 h JA Col-0 replicates was normalized to 1 for each gene, and the ratio was then computed for each specific sample. Our analysis (aggregated profiles, H3K9ac quantification for individual genes and SIENAs, merged replicates for genome browser visualization) excluded the *ninja* 2 h JA replicate 3 and *tpl tpr1 tpr4* WD replicate 1 from the large-scale JA pathway analysis due to insufficient data quality.

#### RNA-seq analysis

RNA-seq sequencing reads were aligned to TAIR10 using the STAR software (version 2.7.5a) (50). Transcripts were first quantified with RSEM (version 1.3.3) and differentially regulated genes were subsequently discovered with edgeR (version 3.36.0) (51). Overall, two sets of RNA-seq experiments were carried out. The first one was conducted for the ctrl and 2 h JA time point of the pilot H3K9ac PHILO ChIP-seq JA time course experiment. Two biological replicates were included in the edgeR analysis which yielded 292 upregulated and 116 downregulated genes (≥4-fold, *P* ≤ 0.05). The second set pertains to the large-scale H3K9ac PHILO ChIP-seq experiment, where gene expression in three biological replicates was assessed for both the ctrl and 2 h JA time points in Col-0 seedlings. The IGV genome browser was used to visualize all sequencing data (52). We used the following three criteria for the selection of MYC2 core genes (Supplementary Table S3): (1) ≥2-fold (*P* ≤ 0.05) JA-induced gene expression (Col-0 ctrl versus 2 h JA, three biological replicates) determined with edgeR (1024 genes), (2) 1000 genes that show the highest JA-inducibility of H3K9ac (Col-0 ctrl vs 2 h JA, three merged biological replicates) determined with epic2 (1010 domains) and (3) genes that show JA-induced MYC2 binding in their regulatory regions (*myc2 MYC2:MYC2-FLAG* ctrl vs 2 h JA, two biological replicates) determined with MACS2 (2980 genes). To identify SIENA regions associated with MYC2 core genes, H3K9ac domains within regulatory regions were initially detected using epic2. Subsequently, these regions were defined as regions that span from the start of the H3K9ac domain to the transcription start site (TSS) of the corresponding MYC2 core gene (Supplementary Table S4).

## Results

To fully understand the function of plant TFs and/or their interactions with the epigenome, complex experimental designs with many variables are required, often resulting in a swift increase in sample quantities that frequently exceed 100 samples (Figure 1A). The current protocols for plant ChIP-seq are inadequate for processing such large sample numbers. To overcome this technical hurdle, we devised the PHILO (Plant HIgh-throughput LOw input) ChIP-seq platform, facilitating the cost-efficient parallel processing of ≥100 samples (Figure 1A). The key advancements over existing methodologies are multifaceted: (1) PHILO ChIP-seq boasts significantly enhanced scalability, (2) it is exceptionally user-friendly and cost-effective, (3) it can accommodate minimal amounts of initial material, such as single *Arabidopsis* seedlings (5–10 mg), 1mg of tissue or 100K nuclei and (4) it provides a liquid handling-only option, expanding the potential user base. This was realized by significantly reducing sample volumes, enabling the utilization of multichannel pipettes with 96-well PCR plates or PCR tube strips and by incorporating a simplified chromatin extraction pipeline. Specifically, the nuclei extraction involving multiple steps and large sample volumes has long hindered efforts to streamline the procedure. However, recently, the eChIP-seq procedure demonstrated a simplification of the nuclei extraction steps (29). This strategy was implemented with modifications into our pipeline. In addition to enhanced scalability, the cost per PHILO ChIP-seq sample is markedly reduced due to the utilization of smaller sample volumes. We significantly reduced amounts of costly consumables such as magnetic protein G/A beads by 80% (from 50 to 10 μl per sample), antibodies by ∼80% (from 5–10 to 0.5–2 μl per sample), protease inhibitors (PIs) by ∼97% (from three pills (50 ml) for three samples to three pills for 100 samples) and library preparation consumables by 50%.

To benchmark our PHILO ChIP-seq pipeline against established methods (regular ChIP-seq, eChIP-seq and CUT&Tag), we analyzed the genome-wide distribution of the histone modification H3K9ac in *Arabidopsis thaliana* wildtype (Col-0) seedlings (Figure 1B, Supplementary Figure S2A–E). The number of identified H3K9ac domains and the reproducibility were highly consistent across replicates of regular ChIP-seq, eChIP-seq and PHILO ChIP-seq (Figure 1B, Supplementary Figure S2B, C). Notably, PHILO ChIP-seq samples demonstrated an improved signal-to-noise ratio, as reflected by higher FRiP scores and better domain enrichment over IgG controls (Supplementary Figure S2B, D). When compared to newly generated H3K9ac CUT&Tag profiles, PHILO ChIP-seq outperformed in terms of the number of identified H3K9ac domains (Figure 1B, Supplementary Figure S2B).

To further assess the quality of PHILO ChIP-seq, we compared four previously published H3K9ac ChIP-seq profiles and one H3K9ac CUT&Tag profile to our PHILO ChIP-seq data (Supplementary Figure S2F–I) (35–39). Only one dataset (38) matched the quality of our PHILO ChIP-seq profiles (Supplementary Figure S2F–I). For a broader comparison between PHILO ChIP-seq, regular ChIP-seq, eChIP-seq and CUT&Tag in plants, we evaluated five key criteria: scalability, cost, low input, time and user-friendliness, using a four-tier rating system (poor, intermediate, good, excellent) (Supplementary Figure S2E). PHILO ChIP-seq clearly performs better than other methods in terms of scalability, cost, low input and user-friendliness (Supplementary Figure S2E).

Moreover, we also successfully profiled H3K4me3-, H3K36me3- and H3K27me3-marked histones with PHILO ChIP-seq in Col-0 seedlings (Figure 1C, Supplementary Figure S2J). In addition to mapping histone modifications, ChIP-seq is extensively employed for capturing global TF binding profiles (53). To validate the utility of PHILO ChIP-seq for analyzing TF binding, we profiled genome-wide DNA binding of the basic loop-helix-loop TF MYC2, the master regulator of the jasmonic acid (JA) pathway (19). Independent PHILO ChIP-seq experiments using untreated and JA-treated (2 h) *myc2 MYC2:MYC2-FLAG* seedlings yielded high-quality datasets that were comparable to previously published MYC2 ChIP-seq data using *MYC2:MYC2-GFP* recombineering lines (Figure 1D) (54). Moreover, our MYC2 PHILO ChIP-seq experiments showed JA-inducibility of MYC2 binding and, as previously reported, the G-box (CACGTG/T) as the preferred DNA-binding motif of MYC2 (Figure 1E, Supplementary Figure S2K) (41,54,55).

**Figure 2.**
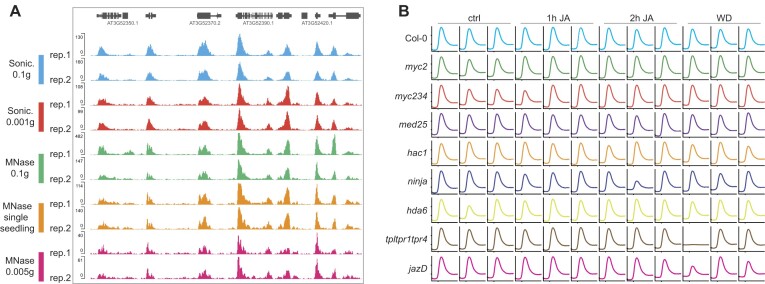
Adaptability of PHILO ChIP-seq. (**A**) Genome browser shows PHILO ChIP-seq-derived H3K9ac occupancy in untreated 10-day-old *Arabidopsis* Col-0 seedling tissue. The indicated fragmentation methods (sonication (Sonic.) and MNase digestion) and varying amounts of starting material were used to generate the displayed H3K9ac profiles. The weight of a single seedling used is approximately 10 mg. All tracks were normalized to their sequencing depth, and two biological replicates are shown. (**B**) Aggregated H3K9ac profiles of 15184 H3K9ac domains derived from a large-scale H3K9ac PHILO ChIP-seq experiment with 108 independent samples (9 genotypes × 4 time points × 3 biological replicates) in total. Three biological replicates are shown for all four time points (ctrl, 1 h, 2 h, 2 h + 2 h JA WD). H3K9ac levels were calculated as the ratio between H3K9ac and IgG control and H3K9ac occupancy is shown from 1 kb upstream to 2 kb downstream of the H3K9ac domain start.

A limiting factor in conducting high-throughput ChIP-seq analyses is the process of chromatin fragmentation, typically achieved through sonication. While 96-well sonicators are available, they remain out of reach for many researchers. To address this, we developed an incremental approach to efficiently fragment over 100 samples in a single PHILO ChIP-seq experiment, utilizing a sonicator capable of handling only 12 samples simultaneously (further details in ‘Material and Methods’ section). Additionally, to expand the accessibility of PHILO ChIP-seq, we introduced an alternative method using MNase digestion for chromatin fragmentation. Comparative analysis of H3K9ac profiles from *Arabidopsis* Col-0 seedlings produced by PHILO ChIP-seq using either sonication or MNase fragmentation revealed similar results (Figure 2A, Supplementary Figure S3A–D).

Timely sample collection and sufficient tissue quantities can present significant challenges for researchers conducting ChIP-seq experiments, making protocols with low input requirements highly desirable. Tissue sampling in large quantities before formaldehyde crosslinking can be labor-intensive and may affect results, particularly in time-sensitive stimulus exposure time-series experiments. In contrast, collecting a single seedling can drastically reduce sampling time. Therefore, we applied PHILO ChIP-seq to single 10-day-old *Arabidopsis* seedlings (10 mg) and successfully produced H3K9ac profiles (Figure 2A, Supplementary Figure S3A–D). Remarkably, even 1 mg of *Arabidopsis* seedling tissue was sufficient to generate high-quality H3K9ac datasets (Figure 2A, Supplementary Figure S3A–D). We also tested the minimum number of nuclei required for successful PHILO ChIP-seq, as rare cell populations are often purified using methods like INTACT (isolation of nuclei tagged in specific cell types) or FANS (fluorescence-activated nuclei sorting), which yield specific numbers of nuclei (56,57). While 100K nuclei yielded high-quality H3K9ac data, the quality significantly dropped when using 20K nuclei (Supplementary Figure S3E–H). Thus, 100K nuclei mark the sensitivity threshold of our PHILO ChIP-seq pipeline. Furthermore, we successfully investigated the H3K4me3 landscape in tomato (*Solanum lycopersicum*) and hemp (*Cannabis sativa*) using PHILO ChIP-seq with MNase-mediated fragmentation (Supplementary Figure S4A–D). Collectively, these findings underscore the adaptability of the PHILO ChIP-seq pipeline across a diverse range of experimental conditions and plant species.

Next, to test PHILO ChIP-seq on a large scale, we investigated JA-induced H3K9ac reprogramming dynamics in *Arabidopsis*. The JA pathway provides an excellent testing ground, given that prevailing models suggest that most identified signaling components carry out their functions by inducing alterations of the histone acetylation landscape at JA-responsive genes (58–60). Intriguingly, bioactive JA-Ile is directly perceived on the chromatin and determines whether the TFs MYC2, MYC3 and MYC4 associate with the histone acetylation (permissive complex) or deacetylation machinery (restrictive complex). In an uninduced state (low levels of bioactive JA), MYC2/3/4 are directly bound by JAZ (JASMONATE-ZIM DOMAIN) proteins, which recruit TPL (TOPLESS)/TPR/HDACs (histone deacetylases) corepressor complexes through the interaction with NINJA (NOVEL INTERACTOR of JAZ) (61,62). In contrast, when bioactive JA accumulates and gets perceived on the chromatin by the JA co-receptor COI1 (CORONATINE INSENSITIVE1)/JAZ complex, JAZ repressors become ubiquitinated and subsequently degraded (63–65). Consequently, previously JAZ-bound MYC2/3/4 are liberated in a JA dose-dependent manner and can associate with the Mediator subunit MED25, HAC1 (HISTONE ACETYLTRANSFERASE OF THE CBP FAMILY1) and the plant Gro/Tup1 family protein LUH (LEUNIG_HOMOLOG) to orchestrate MYC2-dependent gene activation via the acetylation of H3K9 (55,65–66). This elegant scenario has been demonstrated for a limited number of loci. However, the regulatory importance of individual components in shaping the H3K9ac landscape at JA-responsive genes has not been explored (1) in parallel, (2) on a genome-wide scale and (3) in a time course setup. We therefore addressed this gap using our large-scale PHILO ChIP-seq pipeline.

We conducted an initial H3K9ac PHILO ChIP-seq JA time course experiment (ctrl (control), 0.25 h, 0.5 h, 1 h, 2 h, 2 h + 2 h JA WD) to assess the JA-responsiveness of the H3K9ac landscape in Col-0 seedlings. First, we generated transcriptomes using RNA-seq to identify genes that show robust up- and downregulation after 2 h JA (Supplementary Table S2). Then we assessed H3K9ac dynamics over time in these genes and found only marginal differences in H3K9ac levels after 0.25 and 0.5 h JA (Supplementary Figure S4E, F). After 1 h JA, upregulated genes showed a strong increase in H3K9ac, which further increased after 2 h JA (Supplementary Figure S4E, F). Interestingly, JA WD for 2 h led the H3K9ac levels to drop back to ctrl levels (Supplementary Figure S4E, F). We observed the opposite but similar temporal trend for downregulated genes with a smaller dynamic amplitude (Supplementary Figure S4G).

Based on these results, we selected four time points (ctrl, 1 h, 2 h, WD) for our comprehensive JA pathway analysis using PHILO ChIP-seq. In total, nine genotypes (Col-0 + eight mutants) were included in three independent JA time course experiments; *myc2* and *myc2 myc3 myc4* (*myc234*) mutants as master TF mutants, *med25* and *hac1* as permissive complex mutants, and *jazD* (*jaz decuple*), *ninja*, *tpl tpr1 tpr4* and *hda6* as restrictive complex mutants (Supplementary Figure S5A). Around 111 samples (9 genotypes × 4 time points × 3 replicates × 1 mark (H3K9ac) + 3 Col-0 IgG controls) were analyzed in parallel (Figure 2B and Supplementary Figure S5B). From the 111 generated H3K9ac profiles, only 2 samples (*ninja* 2 h JA replicate 2 and *tpl tpr1 tpr4* WD replicate 1) were excluded from our analysis due to low quality (Figure 2B, Supplementary Figure S5B).

**Figure 3.**
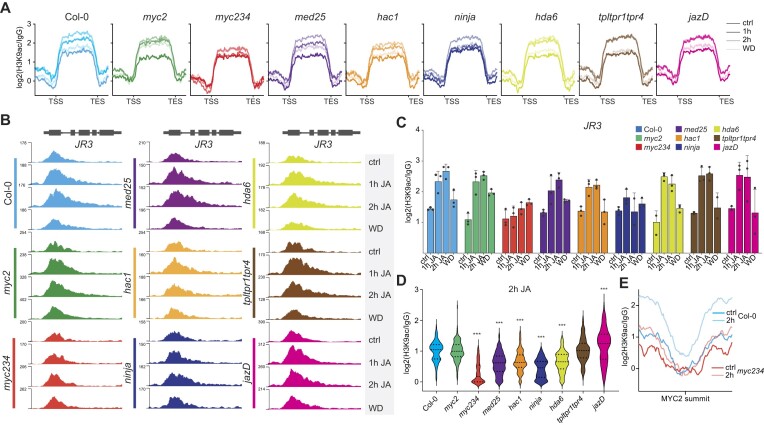
Capture of JA-induced H3K9ac dynamics using PHILO ChIP-seq. **(A)** Metagene plots show JA-induced H3K9ac dynamics of 179 MYC2 core genes in the indicated JA pathway mutants. Plots were derived by merging three biological replicates for each time point and genotype. The 179 MYC2 core genes were scaled to 2 kb and regions 1 kb upstream of the TSS and 0.5 kb downstream of the transcription end site (TES) are shown. Distinct time points (ctrl, 1 h, 2 h, WD) are represented by a color gradient ranging from dark (ctrl) to lightest (WD) for each genotype. (**B**) Genome browser displays JA-regulated H3K9ac dynamics at the *JR3* gene in the indicated mutants. Each track results from merging three biological replicates except for *ninja* 2 h JA and *tpl tpr1 tpr4* WD where only two high-quality replicates were merged. All tracks were normalized to their sequencing depth. (**C**) Quantification of JA-regulated gene body-localized H3K9ac levels at the *JR3* gene in the indicated mutants is shown. H3K9ac levels were calculated as the ratio between H3K9ac and IgG control and the values from each replicate are shown. Error bars indicate standard deviation (SD). (**D**) Violin plot depicts levels of H3K9ac after 2 h JA in the indicated mutants for all 43 SIENA regions. H3K9ac levels were calculated as the ratio between H3K9ac and IgG control. Values for all three replicates at all 43 SIENA regions (129 in total) were included. The average value of all three 2 h JA Col-0 replicates was set to 1 for each gene, and the ratio was calculated for each sample. The statistical significance for differences between Col-0 and respective mutants is denoted by stars (One-way ANOVA, Dunnett's multiple comparisons test, n.s. *P* > 0.05, * *P* ≤ 0.05, ** *P* ≤ 0.01, *** *P* ≤ 0.001). (**E**) H3K9ac profiles in regions spanning 300 bp both upstream and downstream of 60 MYC2 peak summits that were found in 43 SIENA regions are displayed. Profiles are shown for untreated and 2 h JA-treated Col-0 and *myc234* seedlings. Each profile results from merging three biological replicates.

To focus our analysis on genes that are dynamically regulated by JA though MYC2/3/4, we defined the *MYC2 core* gene set as genes that show JA-inducibility (2 h JA) of (1) gene expression, (2) MYC2 binding and (3) gene body-localized H3K9 acetylation. A total of 179 genes fulfilled these three criteria, most of which are known components of the JA and wound signaling pathway (Supplementary Figure S6A–C, Supplementary Table S3). Among them are 9 *JAZ* genes and numerous JA metabolism/catabolism genes (*AOS*, *AOC1-3*, *OPR3*, *LOX2-4*, *JOX2-3*) (Supplementary Table S3). Similar to our initial H3K9ac JA time course (Supplementary Figure S4E–G), MYC2 core genes exhibited the initial measurable elevation of H3K9ac levels after 1 h JA. These levels continued to rise after 2 h JA and reverted to control levels during the WD phase (Figure 3A). Furthermore, these genes displayed a JA-induced augmentation of H3K4me3 and RNAPII (RNA Polymerase II) occupancy, indicating a chromatin environment highly responsive to JA (Supplementary Figure S6D).

Next, we compared gene body-localized H3K9ac dynamics for MYC2 core genes in all nine tested genotypes. After 1 and 2 h of JA, *myc234, hac1, med25, ninja and hda6* mutants exhibited reduced levels of H3K9ac (Figure 3A–C, Supplementary Figure S7A–D). Among these mutants, *myc234* triple mutants showed the most pronounced effect, displaying no changes after 1 h of JA and only a slight increase after 2 h JA (Figure 3A–C, Supplementary Figure S7A–D). This finding is in line with MYC2/3/4′s role as integral recruitment platforms for JA chromatin signaling components (62–66). Our analysis in *hac1* mutants revealed a smaller dynamic amplitude of JA-induced H3K9ac changes at MYC2 core genes which was previously shown for *JAZ8* and *ERF1* (Figure 3A–C, Supplementary Figure S7A–D) (65). *ninja* mutants showed an even smaller dynamic amplitude not only due to slightly higher H3K9ac levels in ctrl plants but also due to lower H3K9ac levels after 1 h JA (Figure 3A–C, Supplementary Figure S7A–D). *med25* and *hda6* mutants displayed only a very mild gene body H3K9ac phenotype when compared to Col-0 (Figure 3A–C, Supplementary Figure S7A–D). In contrast, *tpl tpr1 tpr4* and *jazD* mutants showed a more permissive chromatin environment with H3K9ac levels already reaching their maximum after 1 h JA (Figure 3A–C, Supplementary Figure S7A–D), which supports their roles as negative regulators of the JA pathway (67,68).

Interestingly, we also found a consistent H3K9ac increase in regulatory regions of MYC2 core genes after JA treatment (Figure 3A, Supplementary Figure S6D). Although the levels of H3K9ac were overall lower in regulatory regions, their JA-induced fold change was comparable to that observed in gene bodies after 1 h JA and only slightly lower after 2 h JA (Supplementary Figure S7E). Parallel assessment of JA-induced changes of H3K4me3 levels using PHILO ChIP-seq revealed that JA-induced chromatin changes in regulatory regions are specific to histone acetylation and do not occur for H3K4me3 (Supplementary Figure S6D, Supplementary Figure S7F). Previously, it was demonstrated that various environmental signals, such as low red to far-red light in *Arabidopsis* and heat stress in tomato, can trigger histone acetylation in distant enhancer elements (69,70). We therefore termed these regions, characterized by their swift responsivity to a diverse array of stimuli, as SIENA regions. 43 out of 179 MYC2 core genes were found with a JA-induced H3K9ac enrichment in their regulatory regions (Supplementary Figure S7G and Supplementary Table S4).

**Figure 4.**
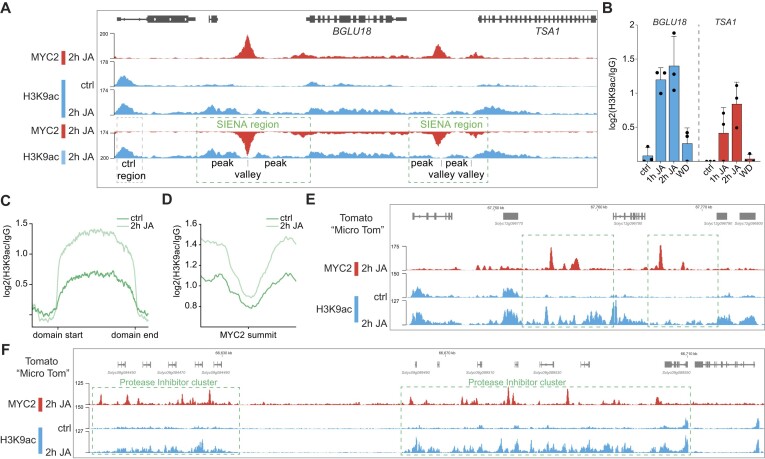
MYC2 shapes SIENA regions in *Arabidopsis* and tomato. **(A)** IGV genome browser tracks show MYC2 binding (2 h JA) and H3K9ac levels (ctrl/ 2 h JA) at the *Arabidopsis BGLU18* and *TSA1* gene. SIENA regions are indicated for both genes. For better visualization of SIENA regions and MYC2-induced valleys within these regions, the MYC2 track was inverted (fourth track). The lower 2 h JA MYC2 and 2 h JA H3K9ac tracks are identical to the upper ones. The respective Col-0 H3K9ac tracks result from merging three biological replicates whereas for 2 h MYC2 replicate 1 is shown. All data results from PHILO ChIP-seq and tracks were normalized to their sequencing depth. (**B**) Quantification of JA-regulated H3K9ac in SIENA regions at the *BGLU18* and *TSA1* gene in *Arabidopsis* Col-0 seedlings. H3K9ac levels were calculated as the ratio between H3K9ac and IgG control. Values from all three replicates are shown, and error bars indicate SD. (**C**) Metagene plots show JA-induced enrichment of H3K9ac at the top 1000 JA differential H3K9ac domains in untreated and 2 h JA-treated Micro-Tom tomato seedlings. The 1000 H3K9ac domains were scaled to 3 kb, and regions 1 kb upstream of the TSS and 0.5 kb downstream of the TES are shown. Three biological H3K9ac PHILO ChIP-seq (MNase fragmentation) replicates were merged for each profile and H3K9ac levels were calculated as the ratio between H3K9ac and IgG control. (**D**) Metagene plots display JA-induced H3K9ac dynamics in regions spanning 500 bp both upstream and downstream of the top 500 MYC2 peak summits in tomato. Profiles are shown for untreated and 2 h JA-treated Micro-Tom tomato seedlings. Each H3K9ac profile results from merging three biological H3K9ac PHILO ChIP-seq (MNase fragmentation) replicates. Relative H3K9ac levels were calculated as the ratio between H3K9ac and IgG control. **(E, F)** Genome browser tracks show tomato MYC2 ChIP-seq (red) and H3K9ac PHILO ChIP-seq (blue) data. SIENA regions at hydroxycinnamoyl CoA quinate transferase genes (*Solyc12g096770*, *Solyc12g096790*, *Solyc12g096800*) (**E**) and in two PI gene clusters (*Solyc09g084450 - Solyc09g084490* and *Solyc09g089490 - Solyc09g089540*) (**F**) are shown. Supplementary Figure S7B shows a subsection of one of the PI gene clusters in higher resolution. SIENA regions are indicated with a green box. MYC2 ChIP-seq data (replicate CRD029134) is derived from Du et al. (41) and all displayed tracks were normalized to their sequencing depth.

When analyzing these SIENA regions in all tested mutants, we again found in *myc234*, *hac1*, *med25*, *ninja* and *hda6* mutants, lower levels of H3K9ac after 1 h and 2 h JA (Figure 3D, Supplementary Figure S7G). This reduction was, however, more pronounced and specifically *med25* mutants showed a stronger H3K9ac phenotype when compared to their minor H3K9ac phenotype in gene bodies (Figure 3D and Supplementary Figure S7G). Interestingly, *JAZ10* expression was previously used to characterize NINJA function (25), however, we observed a unique regulatory phenomenon of *JAZ10* in *ninja* mutants, as it stands out as the sole JAZ gene with a substantial increase in H3K9ac within its SIENA region upon exposure to JA (Supplementary Figure S7H, I). To test whether JA-induced MYC2 DNA binding shapes SIENA regions, we investigated H3K9ac dynamics around MYC2-binding sites. We found a substantial increase in H3K9ac around MYC2-binding sites in SIENA regions upon JA treatment, a distinctive pattern that was markedly impaired in *myc234* mutants and was not evident for H3K4me3 (Figure 3E, Supplementary Figure S7J). By visually inspecting these SIENA regions, we observed characteristic peak-valley-peak patterns where valleys overlap with MYC2-binding sites as exemplified by regulatory regions of *BGLU18*, *TSA1*, *MYC2* and *AOS* (Figure 4A, B, Supplementary Figure S8A–D).

To explore the formation of JA-induced MYC2-dependent SIENAs in other species, we conducted H3K9ac PHILO ChIP-seq analysis in tomato (Micro-Tom variety). Much like its pivotal role in *Arabidopsis*, MYC2 also governs the JA pathway in tomato (41,71,72). Our examination revealed that the H3K9ac profile in tomato exhibits high JA-responsiveness (Figure 4C). Utilizing previously reported MYC2 ChIP-seq datasets (41), we also observed an increase in H3K9ac levels around MYC2-binding sites following JA exposure (Figure 4D). As extensive JA-induced H3K9ac domains spanning from gene bodies to regulatory regions serve as indicators for SIENAs (Figure 4A, B, Supplementary Figure S8A–D), we compared their sizes between *Arabidopsis* and tomato, finding notably larger H3K9ac domains in tomato (average size of the largest 250 H3K9ac domains; 10206 bp in tomato vs 3785 bp in *Arabidopsis*) (Supplementary Figure S9A, Supplementary Table S5). The most extensive JA-induced MYC2-dependent H3K9ac domain we identified in *Arabidopsis* spans 13999 bp, encompassing the *BGLU18* and *TSA1* genes (Figure 4A). Conversely, in tomato, we detected JA-induced H3K9ac domains exceeding 30 kb, stretching across multiple genes and their regulatory regions (Figure 4E, F). Interestingly, the genes within these large clusters encode crucial plant defense components (73,74), such as enzymes (hydroxycinnamoyl CoA quinate transferases) from the phenylpropanoid pathway and PIs (Figure 4E, F). Notably, alongside a substantial number of MYC2-binding sites, SIENAs are also observable in all regulatory regions of genes within these clusters (Supplementary Figure S9B). Furthermore, we also identified SIENAs at individual components of the JA pathway, such as *SlJAZ2* (Supplementary Figure S9C).

## Discussion

The development of PHILO ChIP-seq addresses the limitations of scalability, low input requirements and high costs found in current plant ChIP-seq protocols. Our goal was to create a protocol that allows researchers to perform experiments involving more than 100 samples in a cost-effective way. Large sample numbers are often required when analyzing the occupancies of multiple TFs and/or histone modifications/variants over time, across various genotypes, under different developmental or environmental conditions, with all replicates included. Research areas such as developmental trajectories, TF family studies, complex stimulus interactions, circadian rhythms and natural variation in plants often demand a high number of samples. Currently, most studies on TF binding and chromatin dynamics are conducted in controlled laboratory environments. However, field experiments are becoming increasingly important requiring larger sample numbers due to the need for many replicates at each time point accounting for environmental variability. High-throughput technologies like PHILO ChIP-seq, which facilitate large-scale, rapid and cost-effective experimentation, are essential for accelerating discoveries.

When comparing PHILO ChIP-seq with existing methods like regular ChIP-seq, eChIP and CUT&Tag, PHILO ChIP-seq stands out for its improved scalability, lower costs, minimal starting material requirements and user-friendliness, all while delivering high-quality data. Its user-friendliness, due to a streamlined and cost-efficient protocol where most steps can be done in PCR tubes, makes it accessible to less-experienced researchers. While CUT&Tag can also be used in a high-throughput fashion and at single-cell level in human tissue (8,30,75), this has not yet been demonstrated for plants, so only plant studies were considered in our evaluation. A notable advantage of PHILO ChIP-seq is its adaptability, due to the inclusion of an MNase chromatin fragmentation step, which significantly enhances its multi-omics potential. Currently, PHILO ChIP-seq can capture TF binding and histone modification/variant occupancy, but MNase-fragmented chromatin can also be used to explore other epigenomic features, such as nucleosome positioning with MNase-seq, chromatin accessibility with MH-seq (MNase hypersensitivity sequencing), and three-dimensional chromatin conformation with Micro-C (76–78). Future improvements to the PHILO ChIP-seq pipeline, such as shortening the protocol with Tn5 transposase-mediated tagmentation (as seen in ChIPmentation (79)), could further enhance its utility and decrease its costs.

By applying PHILO ChIP-seq to eight *Arabidopsis* JA pathway component mutants as well as to tomato, we discovered a previously undescribed pattern of H3K9ac around MYC2-binding sites. While the exact function of these SIENA regions remains unknown, a potential role in enhancer activation through chromatin looping can be envisioned. A previous study has demonstrated for a few MYC2 target loci that MYC2-bound MED25 facilitates chromatin looping at these genes, including at *MYC2* itself (55). If H3K9ac indeed mediates and/or marks MED25-induced chromatin loops, it could explain the stronger H3K9ac phenotype of *med25* mutants in SIENA regions (Figure 3D). Very recently, it was also shown with ChIA-PET (Chromatin Interaction Analysis with Paired-End-Tag sequencing) that MYC2 is critical for spatial genome organization in *Arabidopsis* (80). Our PHILO ChIP-seq-mediated pathway profiling approach not only supported and expanded the existing comprehension of the MYC2 module but also provided new insights into the JA-induced and MYC2/3/4-dependent formation of SIENAs. The discovery of SIENAs within extensive defense gene clusters in tomato not only suggests the conservation of histone acetylation around MYC2-binding sites across various plant species but also implies that SIENAs could serve as markers for the existence of loop structures facilitating TFs. Despite detecting altered amplitudes of H3K9ac dynamics in most tested JA pathway mutants, the overall pattern remained robust, except for *myc234* mutants. This suggests the presence of additional, as-yet-unknown components contributing to the JA-responsive chromatin landscape. Although *NINJA* is unlike most other tested pathway components a single copy gene, its H3K9ac phenotype is surprising given its role as a negative regulator serving as a bridge between JAZ repressors and TPL/TPR proteins (62).

In conclusion, by extensively mapping the H3K9ac landscape throughout an entire plant hormone signaling pathway, our study showcases the effectiveness of PHILO ChIP-seq for conducting large-scale chromatin dynamics research.

## Supplementary Material

gkae1123_Supplemental_Files

## Data Availability

All ChIP-seq, PHILO ChIP-seq and RNA-seq data can be accessed at GEO (GSE249738).
