# Supplementary material for: High-throughput capture of transcription factor-driven epigenome dynamics using PHILO ChIP-seq
Source: Nucleic Acids Res. 2024 Nov 26;52(22):e105. doi: 10.1093/nar/gkae1123 (PMC11662648; doi:10.1093/nar/gkae1123)
Supplement: gkae1123_Supplemental_Files [file gkae1123_supplemental_files.zip › Supplementary Data.pdf]

## SUPPLEMENTARY DATA

# High-throughput capture of transcription factor-driven epigenome dynamics using PHILO ChIP-seq

Aanchal Choudhary[1, #], Moonia Ammari[1, #], Hyuk Sung Yoon[1], and Mark Zander[1, *]

## Figure S1

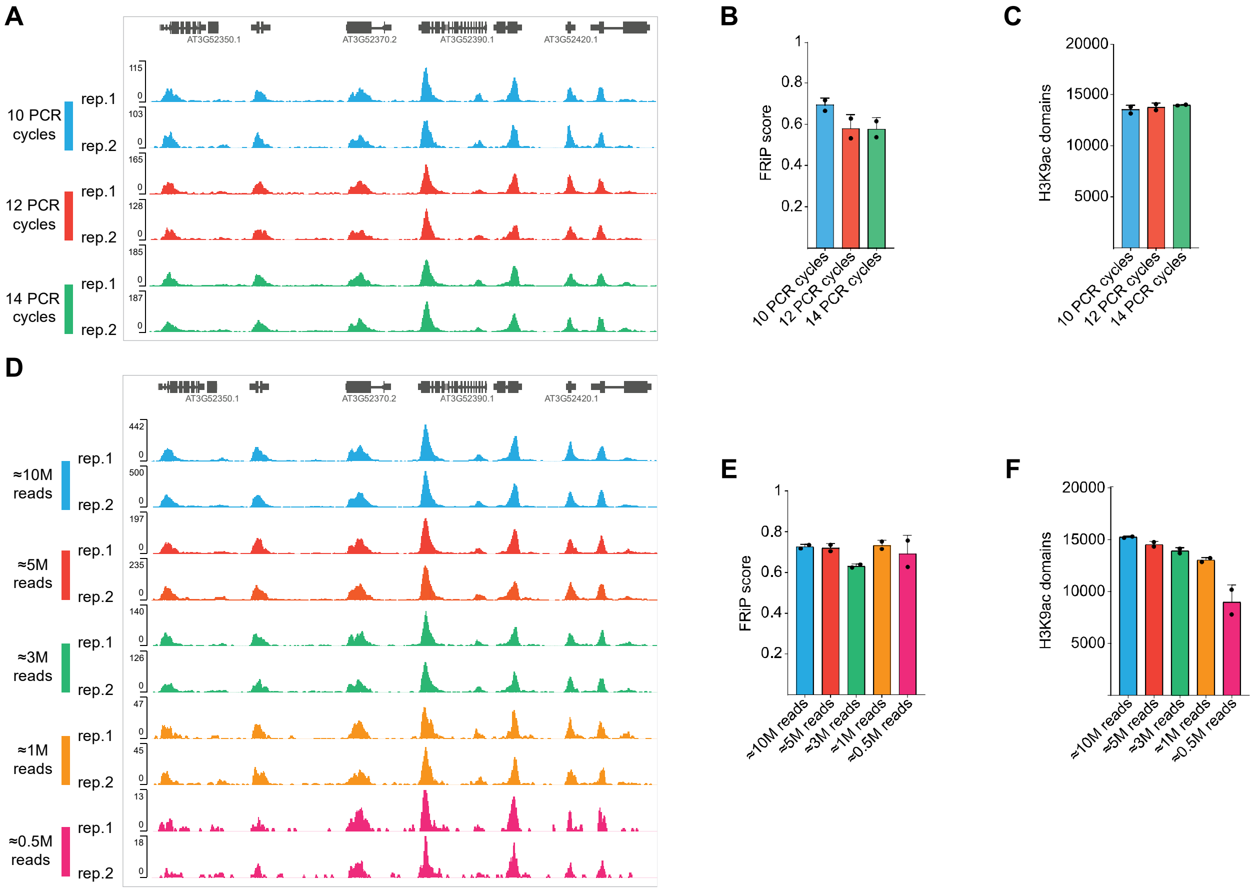


**Supplementary Figure S1. Impact of PCR cycle number and sequencing depth on PHILO ChIP-seq data quality**

**A,** Genome browser shows H3K9ac occupancy in untreated 10-day-old *Arabidopsis* Col-0 seedlings determined by PHILO ChIP-seq using sequencing libraries that were generated with the indicated PCR cycle numbers. All samples were independently processed, and the PCR enrichment step during library preparation was carried out with the indicated PCR cycle numbers. **B, C,** FRiP scores (**B**) and respective number of H3K9ac domains (**C**) are shown. **D,** Genome browser shows PHILO ChIP-seq-derived H3K9ac occupancy in untreated 10-day-old *Arabidopsis* Col-0 seedlings at the indicated sequencing depths. All samples were independently processed and sequenced to the indicated depths. Approximate sequencing depths are shown, and exact depths are listed in Supplementary Table 1. **E, F,** FRiP scores (**E**) and respective number of H3K9ac domains (**F**) are shown for each replicate. Error bars indicate SD.

**Figure S2**

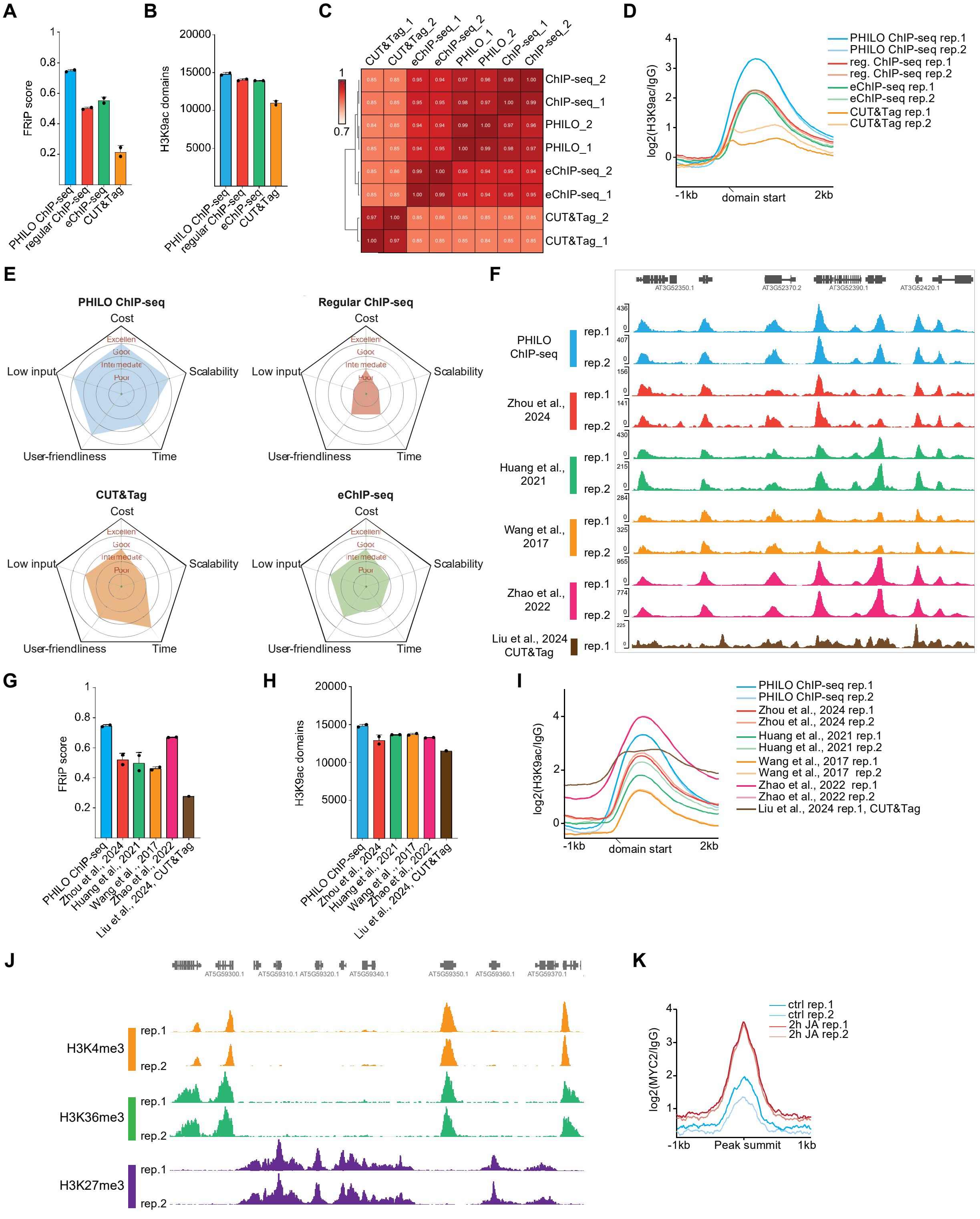


**Supplementary Figure S2. PHILO ChIP-seq generates high-quality datasets.**

**A, B,** FRiP scores (**A**) and number of H3K9ac domains (**B**) in the individual regular ChIP-seq, eChIP-seq, CUT&Tag, and PHILO ChIP-seq replicates are shown. **C,** Spearman's correlation plot shows the correlation between all indicated replicates in 10569 H3K9ac domains. The degree of

correlation determines the clustering. **D,** Aggregated profiles visualize H3K9ac occupancy of 10569 domains in two replicates of each method. Regions from 1 kb upstream to 2 kb downstream of the H3K9ac domain start are shown. **E,** Comparison between methods with respect to five specific features (cost, scalability, time, user-friendliness, and low input). **F,** Genome browser shows H3K9ac levels in two PHILO ChIP-seq replicates, four published H3K9ac ChIP-seq datasets, each containing two replicates and one published H3K9ac CUT&Tag dataset. All shown samples are derived from *Arabidopsis* Col-0 tissue. **G, H,** FRiP scores (**G**) and respective numbers of H3K9ac domains (**H**) are shown for each replicate. Error bars indicate SD. **I,** Aggregated profiles visualize H3K9ac occupancy for all indicated datasets. Regions from 1 kb upstream to 2 kb downstream of the H3K9ac domain start are shown. **J,** Genome browser shows an example region with H3K4me3-, H3K36me3-, and H3K27me3-marked histones in two PHILO ChIP-seq replicates derived from untreated *Arabidopsis* Col-0 seedlings. All tracks were normalized to their sequencing depth. **K,** Aggregated profiles of the 500 top MYC2 targets show JA-inducibility of MYC2 binding. Displayed are the profiles of two biological MYC2 PHILO ChIP-seq in regions spanning 1 kb both upstream and downstream of the top 500 MYC2 peak summits. Data is derived from PHILO ChIP-seq using *myc2 MYC2:MYC2-FLAG* seedlings.

**Figure S3**

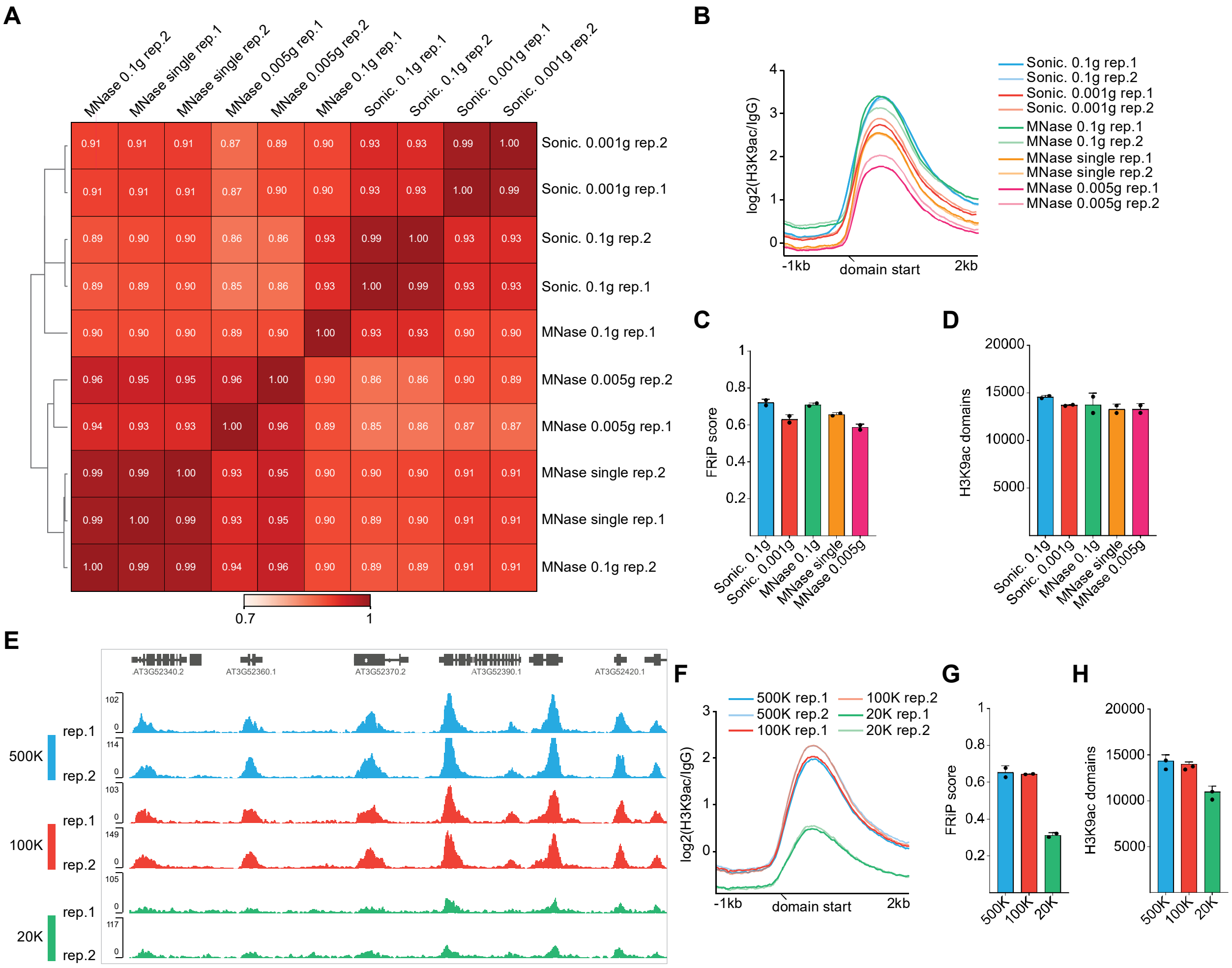


**Supplementary Figure S3. PHILO ChIP-seq is highly versatile.**

**A,** Spearman's correlation plot shows correlation in 10569 H3K9ac domains between H3K9ac PHILO ChIP-seq profiles derived from indicated fragmentation methods (sonication (Sonic.) and MNase digestion) with varying amounts of starting material. Two biological replicates for each of the five treatment combinations are shown. Single seedlings (Single) weigh approximately 10mg. **B,** Aggregated profiles visualize H3K9ac occupancy of 10569 domains in the respective PHILO ChIP-seq samples with the indicated treatment combinations. Regions from 1 kb upstream to 2 kb downstream of the H3K9ac domain start are shown. **C, D,** FRiP scores (**C**) and number of H3K9ac domains (**D**) identified with the indicated treatment combinations are shown. **E,** Genome browser shows H3K9ac occupancy in PHILO ChIP-seq samples that were derived from the indicated numbers of *Arabidopsis* Col-0 nuclei. All tracks were normalized to their sequencing depth. **F,** Aggregated profiles visualize H3K9ac occupancy in indicated replicates. Regions from 1 kb upstream to 2 kb downstream of the H3K9ac domain start are shown. **G, H,** FRiP scores (**G**) and respective number of H3K9ac peaks (**H**) are shown for each replicate. Error bars indicate SD.

**Figure S4**

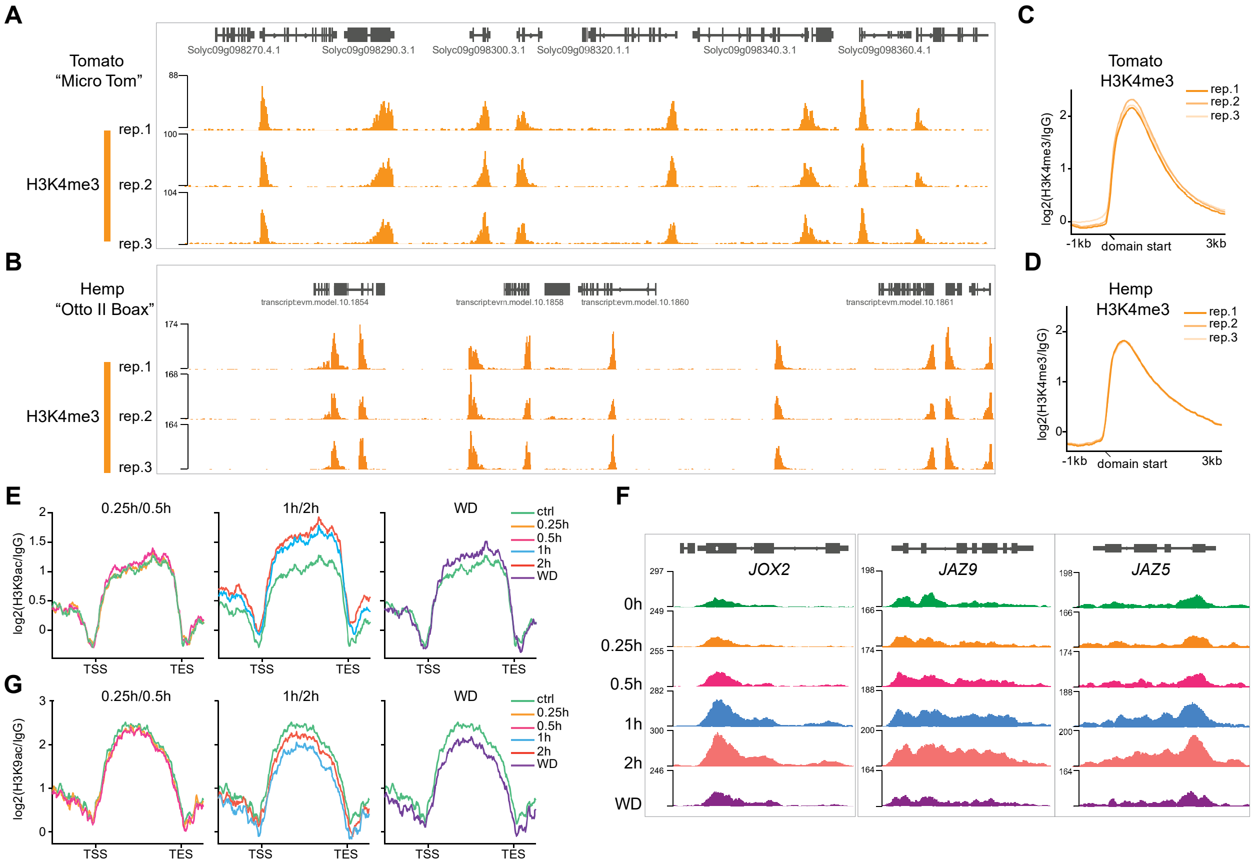


**Supplementary Figure S4. PHILO ChIP-seq can be applied to crop species.**
**A, B,** Genome browser shows an example region with H3K4me3-marked histones in three PHILO ChIP-seq replicates derived from untreated two-week-old Micro-Tom tomato seedlings (**A**) and Otto II Baox hemp seedlings (**B**). All tracks were normalized to their sequencing depth and genomic regions are indicated. **C, D,** Aggregated profiles visualize H3K4me3 occupancy of 18163 domains in tomato (**C**) and 14960 domains in hemp (**D**). Regions from 1 kb upstream to 3 kb downstream of the H3K4me3 domain start are shown. **E,** Metagene plots show JA-induced H3K9ac enrichment in JA-upregulated (292 genes) genes during a JA H3K9ac PHILO ChIP-seq time course experiment (ctrl, 0.25 h, 0.5 h, 1 h, 2 h, 2 h + 2 h JA withdrawal (WD)). For better visualization, time points were separated into three different plots (0.25 h/0.5 h, 1 h/ 2 h, WD). H3K9ac levels were calculated as the ratio between H3K9ac and IgG control. JA-upregulated genes were scaled to 2 kb, and regions 1 kb upstream of the TSS and 0.5 kb downstream of the TES are shown. **F,** Genome browser shows H3K9ac dynamics over time at *JOX2*, *JAZ9* and *JAZ5*. All tracks were normalized to their sequencing depth. **G,** Metagene plots show JA-induced H3K9ac enrichment in JA-downregulated (116 genes) genes during a JA H3K9ac PHILO ChIP-seq time course experiment (ctrl, 0.25 h, 0.5 h, 1 h, 2 h, The ctrl profile (green) is identical in all three plots for upregulated and downregulated genes. JA-downregulated genes were scaled to 2 kb, and regions 1 kb upstream of the TSS and 0.5 kb downstream of the TES are shown. All data is derived from Col-0 seedlings, and three biological replicates were merged for each profile.

## Figure S5

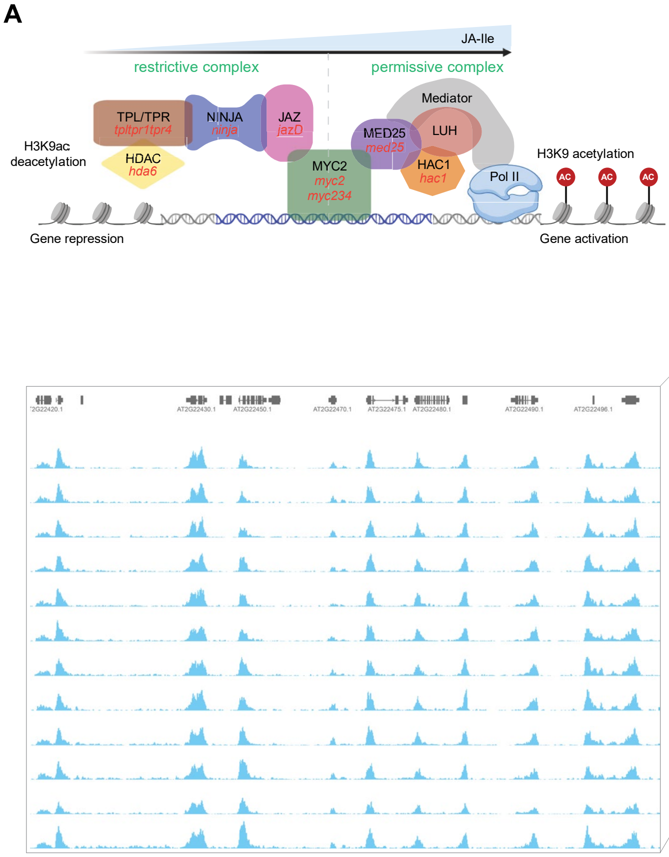
A
JA-Ile
restrictive complex
permissive complex
TPL/TPR
tpltpr1tpr4
NINJA
ninja
JAZ
jazD
Mediator
MED25
med25
LUH
H3K9ac
deacetylation
HDAC
hda6
MYC2
myc2
myc234
HAC1
hac1
Pol II
H3K9 acetylation
AC
AC
AC
Gene repression
Gene activation
AT2G22430.1
AT2G22450.1
AT2G22470.1
AT2G22475.1
AT2G22480.1
AT2G22490.1
AT2G22496.1

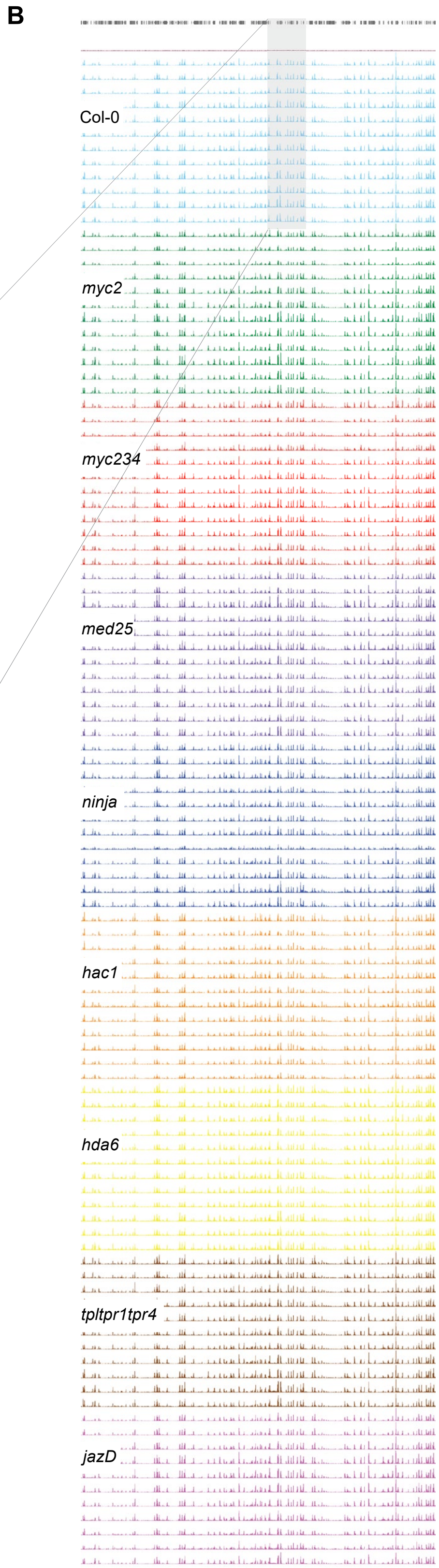
B
Col-0
myc2
myc234
med25
ninja
hac1
hda6
tpltpr1tpr4
jazD

**Supplementary Figure S5. PHILO ChIP-seq-mediated large-scale assessment of chromatin dynamics.**

**A,** Schematic overview of the MYC2 core module and its critical components under low levels (restrictive complex) and high levels (permissive complex) of JA-Ile. Mutants used in this study are indicated in red. Illustration was generated with BioRender. **B,** Genome browser shows PHILO ChIP-seq H3K9ac tracks of 108 samples derived from 9 genotypes, each with 12 samples (3 x ctrl, 1 h, 2 h, WD). The tested mutants are indicated, and the Col-0 tracks are enlarged for better quality assessment.

## Figure S6

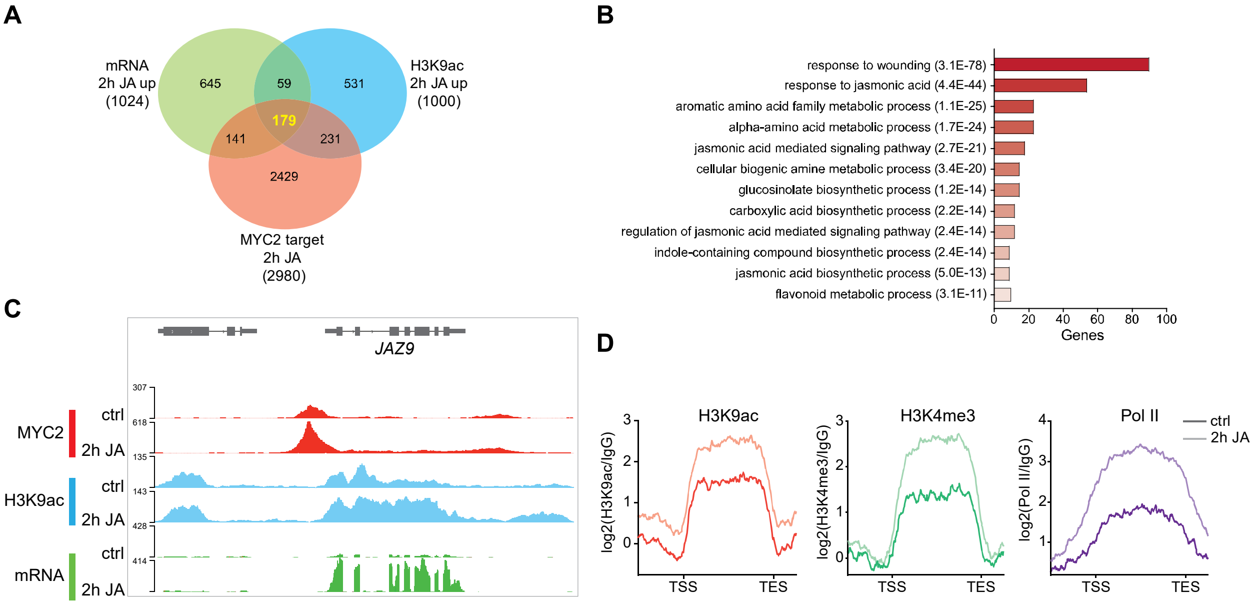


**Supplementary Figure S6. Defining the MYC2 core gene set.**
**A,** Venn diagram shows an overlap between genes that show JA-induced gene expression (2 h JA, 1024 genes), JA-induced H3K9ac enrichment (2 h JA, 1000 genes) and JA-induced MYC2 binding (2 h JA, 2980 genes). 179 genes met all three criteria and thus represent the MYC2 core gene set (indicated in yellow). **B,** Gene ontology analysis discovered the wound and JA signalling pathway as the major categories within the MYC2 core gene set. Enrichment P-values are shown for each category. **C,** Genome browser shows *JAZ9* as a MYC2 core gene example. H3K9ac and MYC2 data are derived from PHILO ChIP-seq of Col-0 and *myc2 MYC2:MYC2-FLAG* seedlings, respectively, and gene expression is derived from Col-0 seedlings using RNA-seq. MYC2 and mRNA tracks show only one representative replicate, whereas the H3K9ac tracks are derived from three merged biological replicates. All tracks were normalized within the same feature to their sequencing depth. **D,** Metagene plots show JA-induced enrichment of H3K9ac, H3K4me3, and RNAPII at 179 MYC2 core genes in untreated and 2 h JA-treated Col-0 seedlings. The 179 MYC2 core genes were scaled to 2 kb and regions 1 kb upstream of the TSS and 0.5 kb downstream of the TES are shown. Three biological replicates were merged for each profile and H3K9ac levels were calculated as the ratio between H3K9ac and IgG control.

**Figure S7**

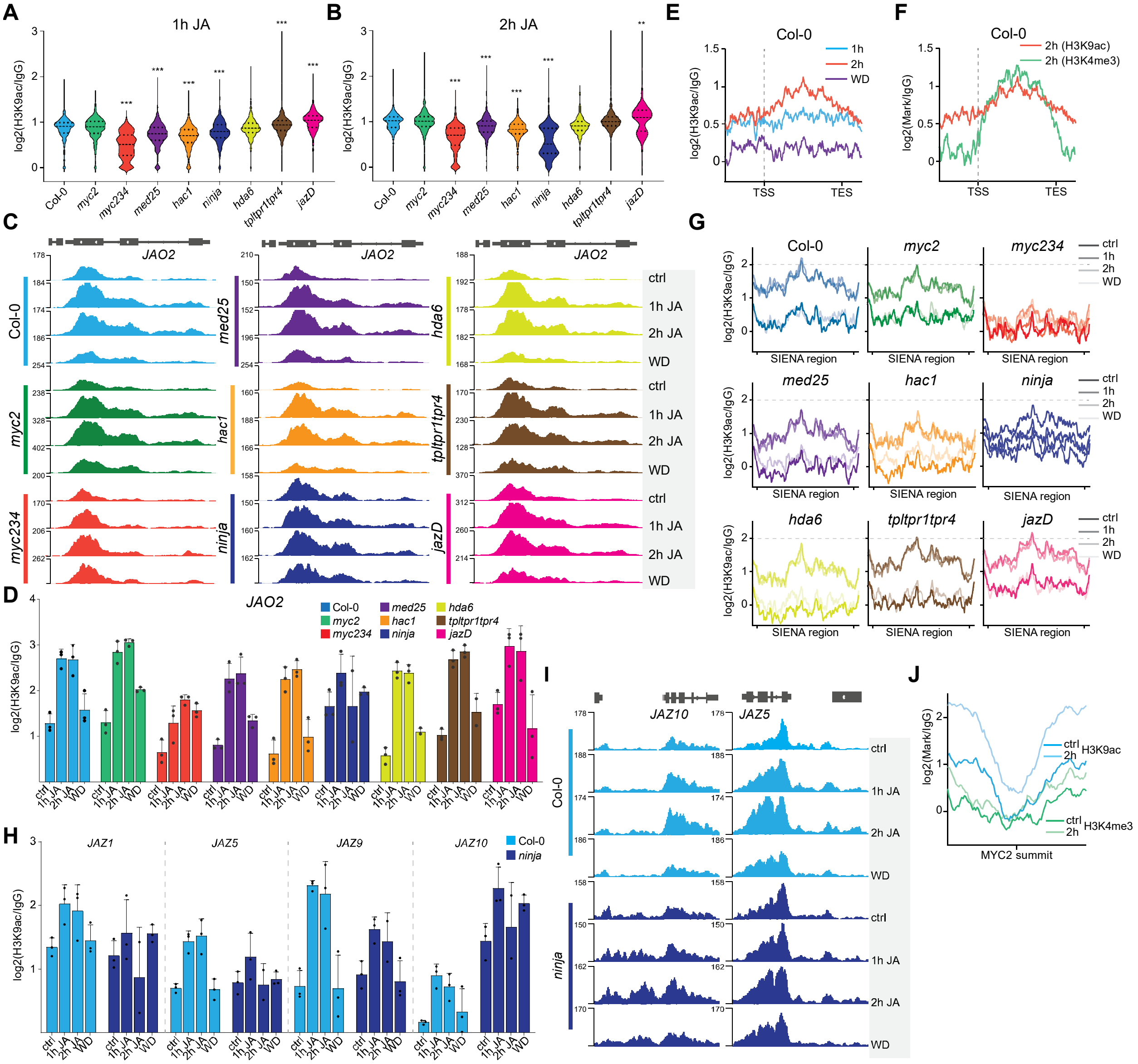


**Supplementary Figure S7. Assessing the regulatory contribution of JA pathway mutants.** **A, B,** Violin plot depicts levels of H3K9ac in gene bodies after 1 h (**A**) and 2 h (**B**) JA in the indicated mutants in 179 MYC2 core genes. H3K9ac levels were calculated as the ratio between H3K9ac and IgG control. Values for all three replicates at all 179 MYC2 core genes (537 in total) were included. The average value of all three 2 h JA Col-0 replicates was set to 1 for each gene and the ratio was calculated for each sample. The statistical significance for differences between Col-0 and respective mutants is denoted by stars (One-way ANOVA, Dunnett's multiple comparisons test, n.s. P > 0.05, * P ≤ 0.05, ** P ≤ 0.01, *** P ≤ 0.001). **C,** Genome browser shows JA-regulated H3K9ac dynamics at the *JAO2* gene in the indicated mutants. Each track results from merging three biological replicates except for *ninja* 2 h JA and *tpltrp1trp4* WD where only two high-quality replicates were merged. All tracks were normalized to their sequencing depth. **D,** Quantification of JA-regulated gene body-localized H3K9ac levels at the *JAO2* gene in the indicated mutants. H3K9ac levels were calculated as the ratio between H3K9ac and IgG control and the values from each replicate are shown. Error bars indicate SD. **E, F,** Metagene plot shows

JA-induced fold change of H3K9ac (**E**) and H3K4me3 (**F**) at 179 MYC2 core genes in untreated and JA-treated (1 h, 2 h, WD) Col-0 seedlings. Three biological replicates were merged for each profile. Fold change of H3K9ac was calculated as the ratio between H3K9ac (1 h or 2 h) and H3K9ac ctrl. Fold change of H3K4me3 was calculated as the ratio between H3K4me3 (2 h) and H3K4me3 ctrl. The 179 MYC2 core genes were scaled to 2 kb and regions 1 kb upstream of the TSS and 0.5 kb downstream of the TES are shown. The 2 h JA H3K9ac profile (red) is identical in E and F. **G,** Metagene plots show JA-induced H3K9ac dynamics of 43 SIENA regions in the indicated JA pathway mutants. Plots were derived by merging the three biological replicates for each time point and genotype. The 43 SIENA genes were scaled to 2 kb and regions 1 kb upstream of the TSS and 0.5 kb downstream of the TES are shown. Grey dashed line indicates log2 fold change. Distinct time points (ctrl, 1 h, 2 h, WD) are represented by a color gradient ranging from dark (ctrl) to lightest (WD) for each genotype. **H,** Quantification of H3K9ac levels in SIENA regions at the indicated *JAZ* genes revealed a stark increase of H3K9ac in *ninja* mutants only for *JAZ10*. Levels were calculated as the ratio between H3K9ac and IgG control. Values from all three replicates except for *ninja* 2 h JA with two replicates are shown and error bars indicate SD. **I,** Genome browser shows H3K9ac dynamics at *JAZ5* and *JAZ10* in Col-0 and *ninja* seedlings. Each track results from merging three biological H3K9ac PHILO ChIP-seq. All tracks were normalized to their sequencing depth. **J,** Metagene plots display JA-induced H3K9ac and H3K4me3 dynamics in regions spanning 300 bp both upstream and downstream of 60 MYC2 peak summits that were found in 43 SIENA regions. Profiles are shown for untreated and 2 h JA-treated Col-0 seedlings derived from merging three biological replicates. The ctrl and 2 h JA H3K9ac profiles are identical to the profiles in Figure 3E.

**Figure S8**

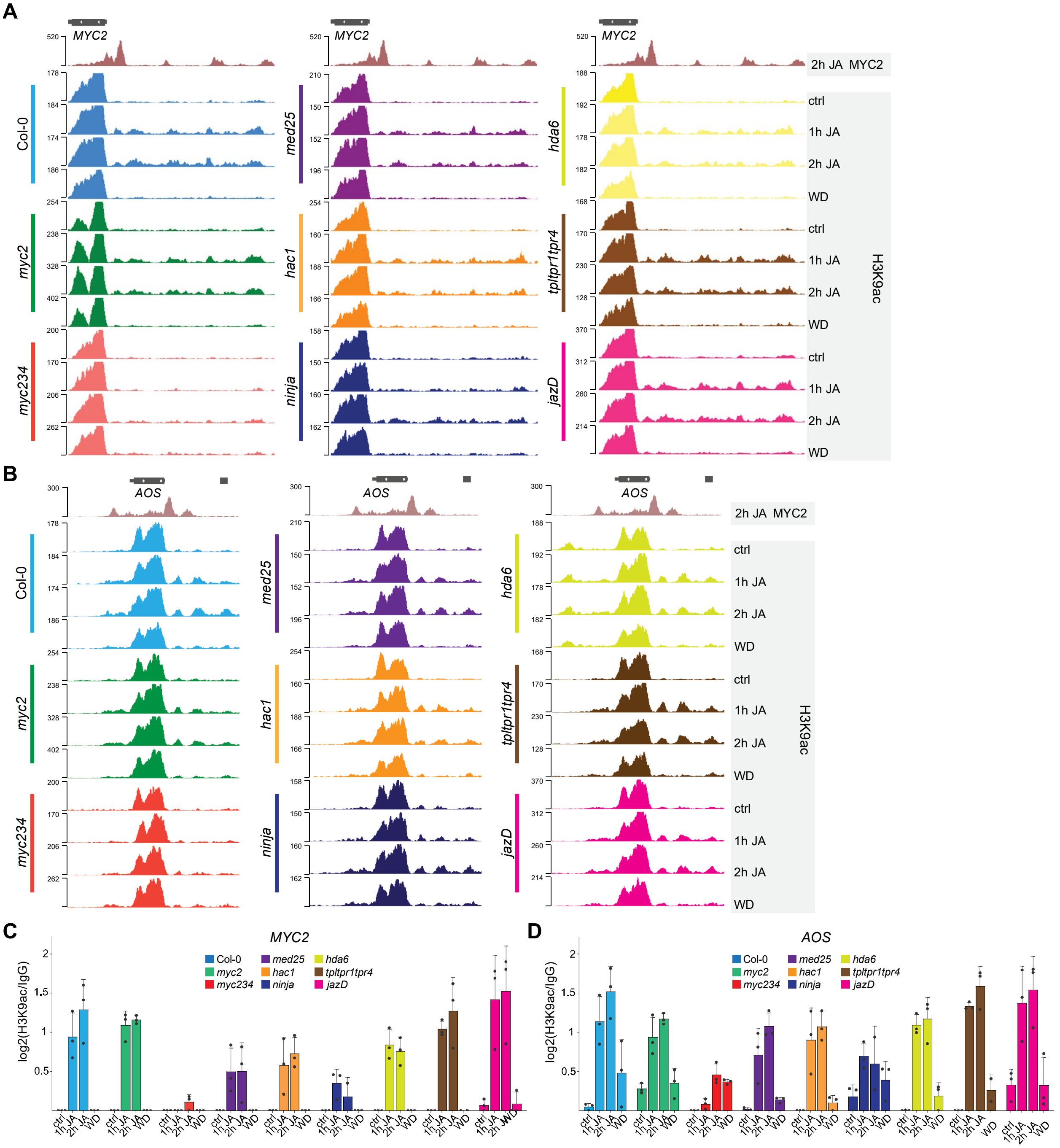


**Supplementary Figure S8. Example of large SIENA regions in *Arabidopsis*.**

**A, B,** Genome browser shows the *MYC2* (**A**) and *AOS* (**B**) genes as examples to illustrate JA-induced formation of SIENAs in the indicated genotypes. Each track results from merging three biological H3K9ac PHILO ChIP-seq. MYC2 binding after 2 h JA is also shown in the first track of each subpanel. All tracks were normalized to their sequencing depth. **C, D,** Quantification of JA-regulated H3K9ac in SIENA regions at the *MYC2* (**C**) and *AOS* (**D**) gene in indicated JA pathway mutants. H3K9ac levels were calculated as the ratio between H3K9ac and IgG control. Values from all three replicates are shown and error bars indicate SD.

**Figure S9**

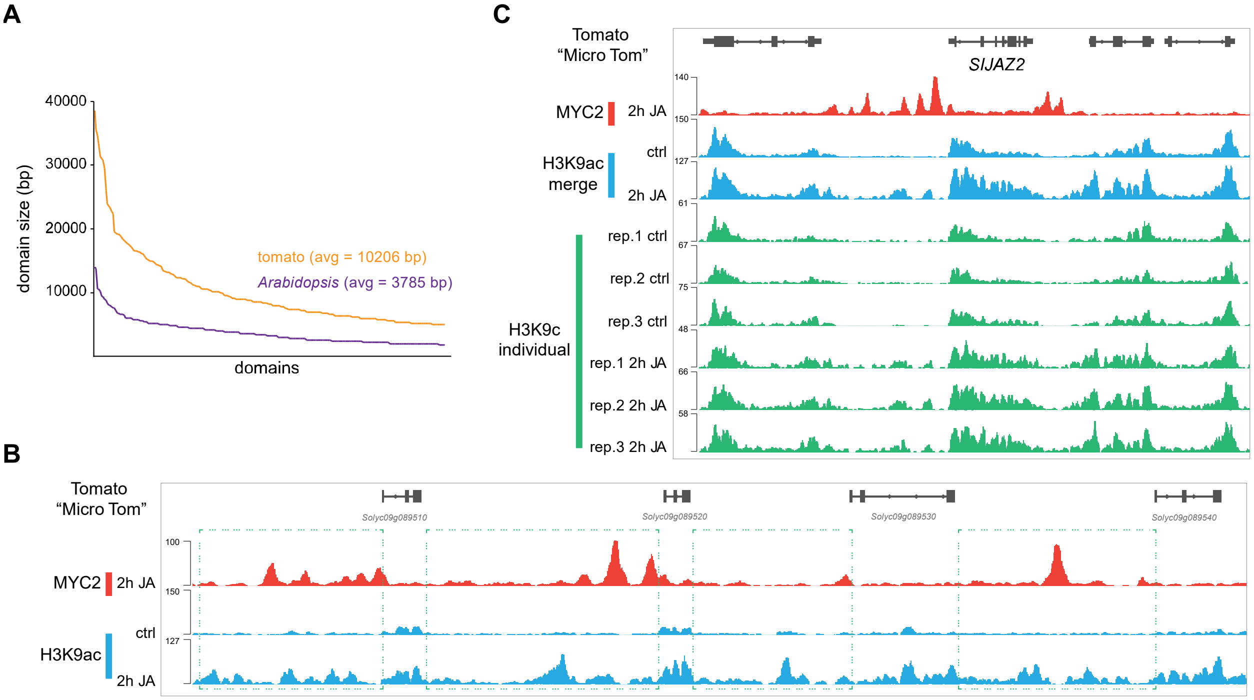


**Supplementary Figure S9. SIENA regions in tomato.**

**A,** Size distribution plot shows sizes of the 250 largest JA-induced H3K9ac domains in *Arabidopsis* and tomato. Average (avg) sizes for each species are indicated. **B,** Genome browser tracks show MYC2 binding and H3K9ac occupancy at SIENA regions of PI genes. These PI genes belong to the bigger PI gene cluster shown in Figure 4F. SIENA regions are indicated with a green box. All tracks were normalized to their sequencing depth. **C,** Genome browser tracks show MYC2 and H3K9ac occupancy at the tomato *SlJAZ2* gene. H3K9ac data is derived from H3K9ac PHILO ChIP-seq (MNase fragmentation) experiments. Individual as well as merged replicates are shown. All tracks were normalized to their sequencing depth. MYC2 ChIP-seq data in **B**, **C** (replicate CRD029134) is derived from Du et al., 2017.
